# Supplementary figures and images for: RNA Polymerase II transcription independent of TBP in murine embryonic stem cells
Source: eLife. 2023 Mar 30;12:e83810. doi: 10.7554/eLife.83810 (PMC10174690; doi:10.7554/eLife.83810)

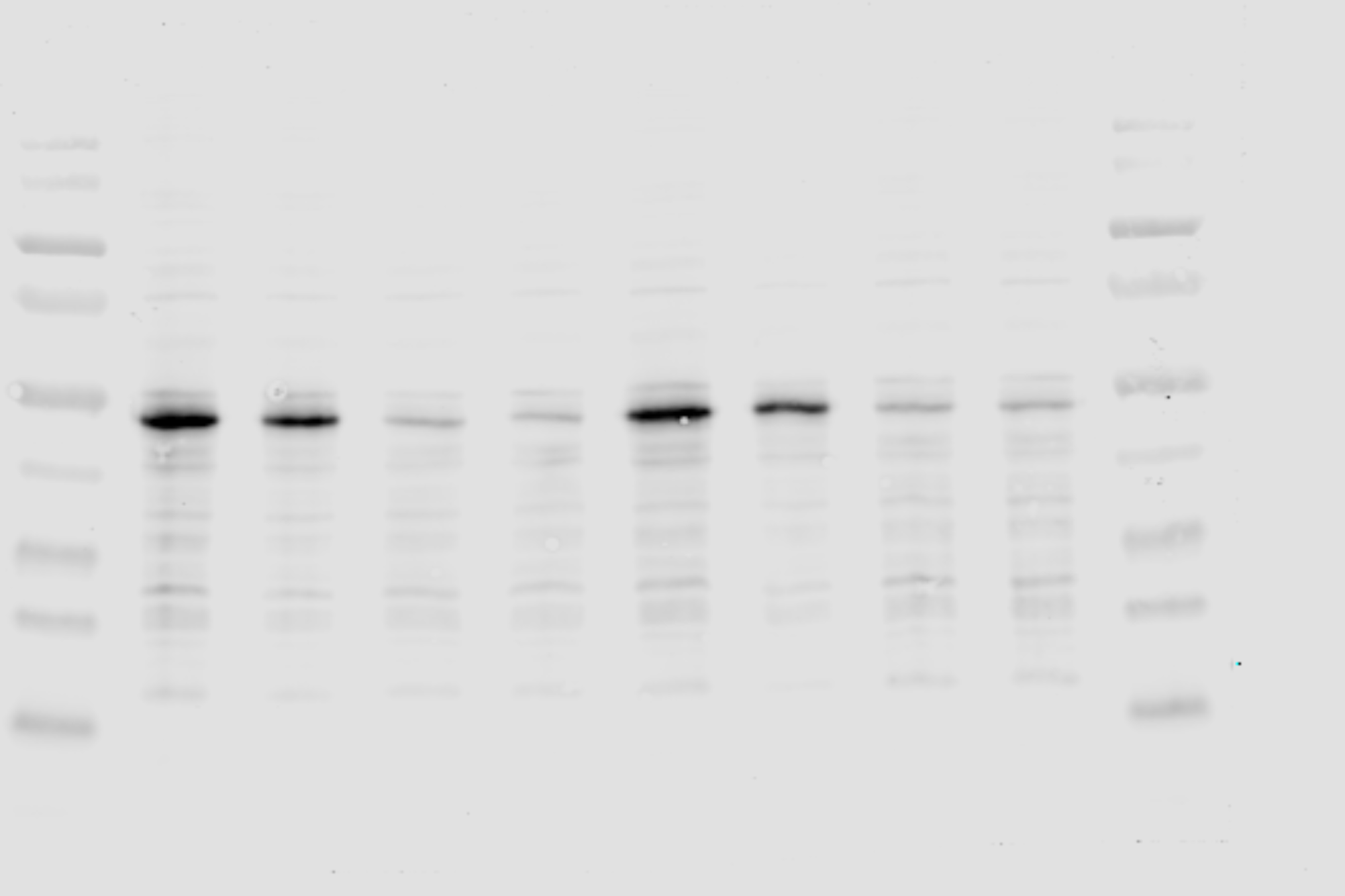

Supplement: Figure 1—source data 1. — Relevant bands for Figure 1B are highlighted with a red rectangle. L=ladder, x=lanes not used in Figure 1B. [file elife-83810-fig1-data1.zip › Figure 1-source data 1/Figure 1-source data 1 TBP raw.tif]

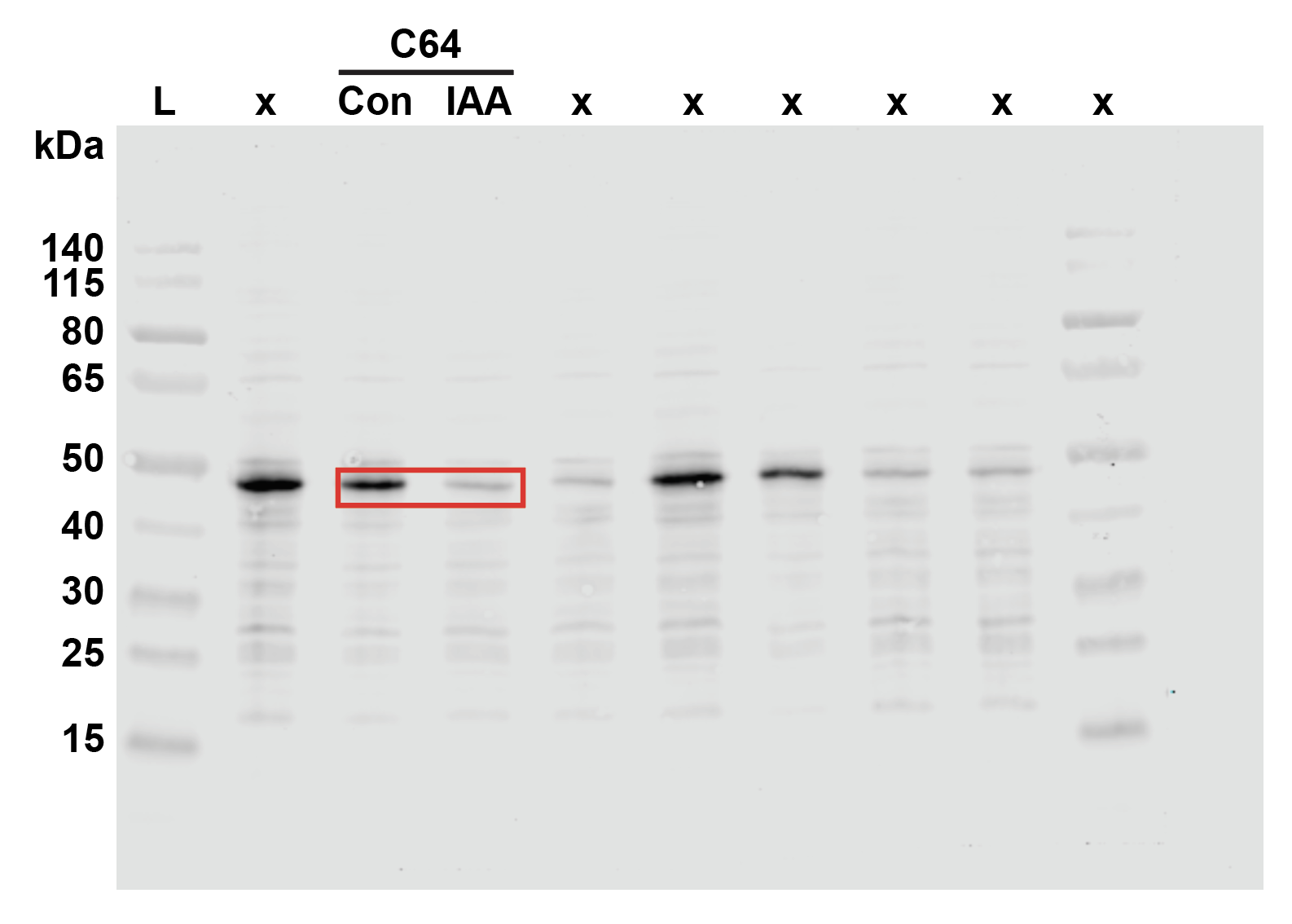

Supplement: Figure 1—source data 1. — Relevant bands for Figure 1B are highlighted with a red rectangle. L=ladder, x=lanes not used in Figure 1B. [file elife-83810-fig1-data1.zip › Figure 1-source data 1/Figure 1-source data 1 TBP annotated.tiff]

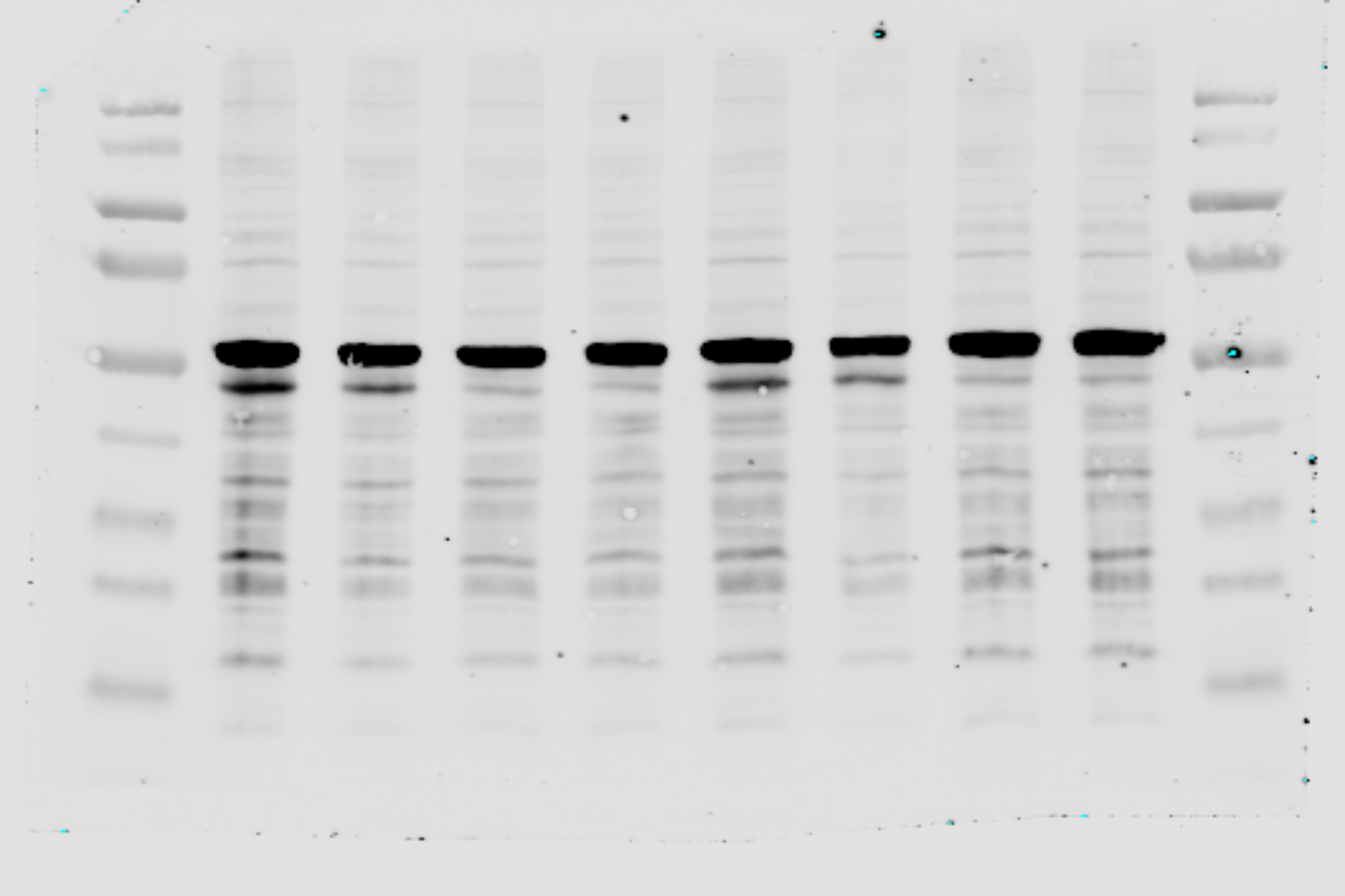

Supplement: Figure 1—source data 2. — Relevant bands for Figure 1B are highlighted with a red rectangle. L=ladder, x=lanes not used in Figure 1B. [file elife-83810-fig1-data2.zip › Figure 1-source data 2/Figure 1-source data 2 Tubulin raw.tif]

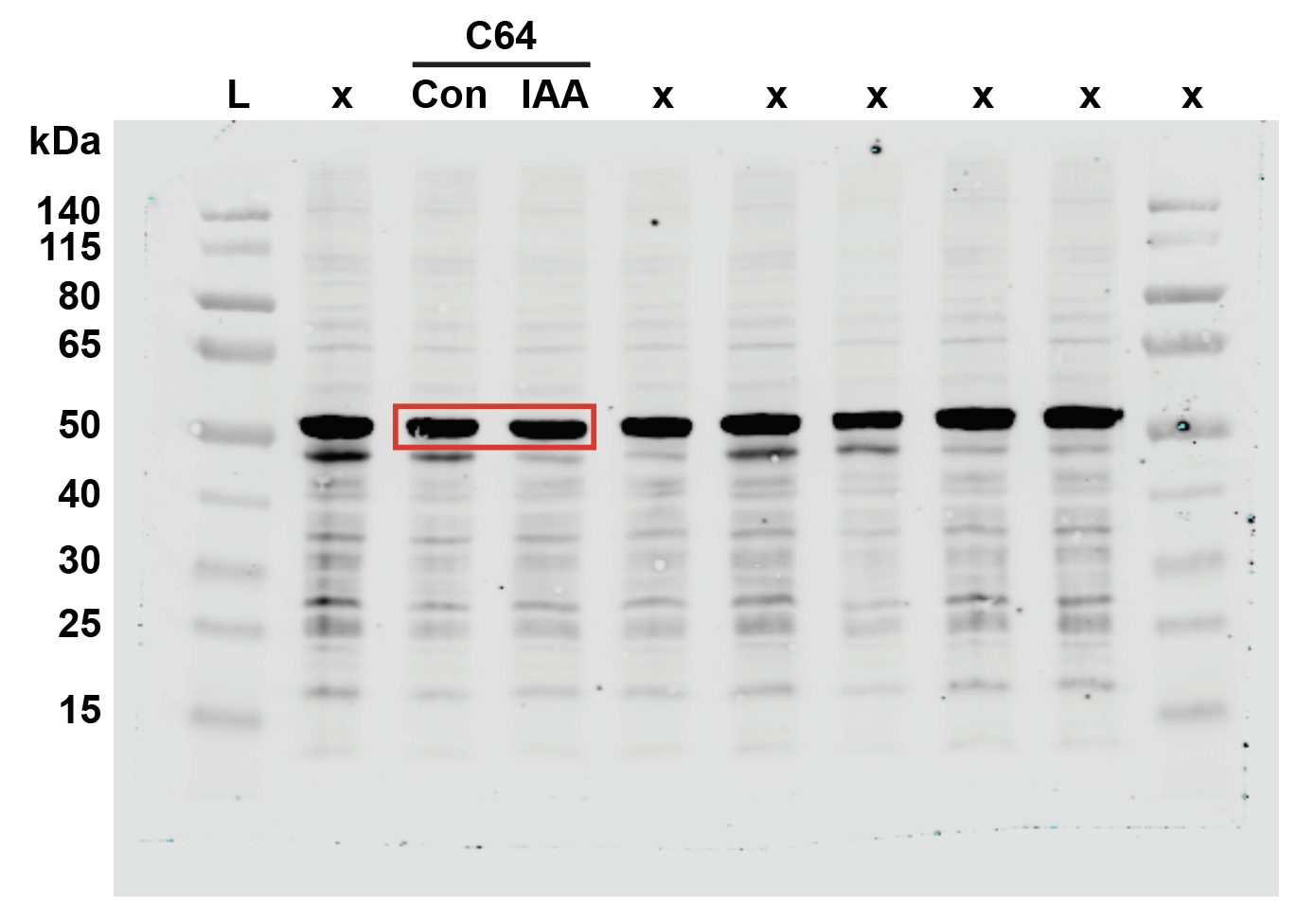

Supplement: Figure 1—source data 2. — Relevant bands for Figure 1B are highlighted with a red rectangle. L=ladder, x=lanes not used in Figure 1B. [file elife-83810-fig1-data2.zip › Figure 1-source data 2/Figure 1-source data 2 Tubulin annotated.tiff]

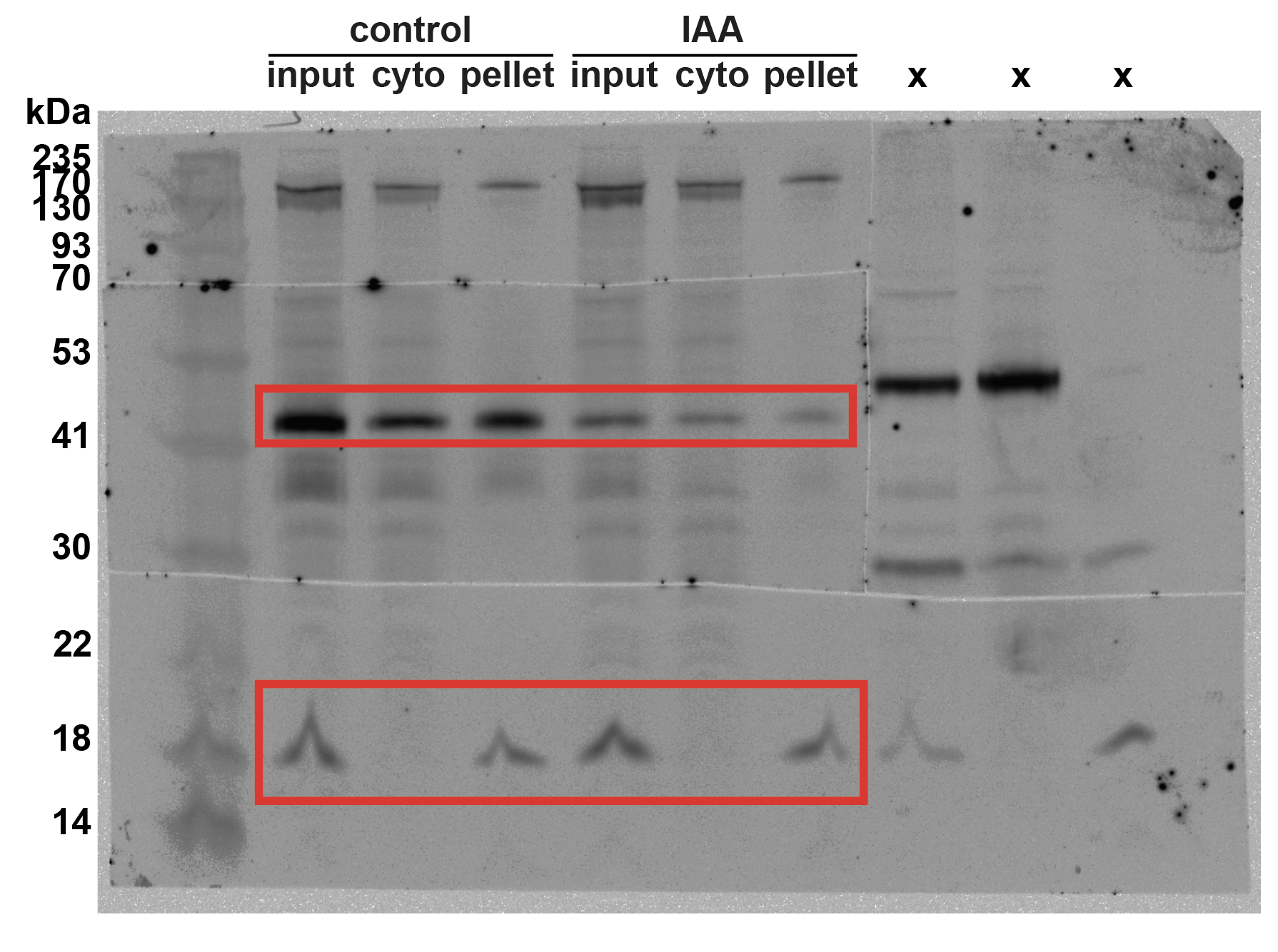

Supplement: Figure 1—figure supplement 1—source data 1. — Relevant bands for Figure 1—figure supplement 1C are highlighted with a red rectangle. L=ladder, x=lanes not used in Figure 1—figure supplement 1C. [file elife-83810-fig1-figsupp1-data1.zip › Figure 1-figure supplement 1-source data 1/Figure 1-figure supplement 1-source data 1 TBP H3.3 annotated.tiff]

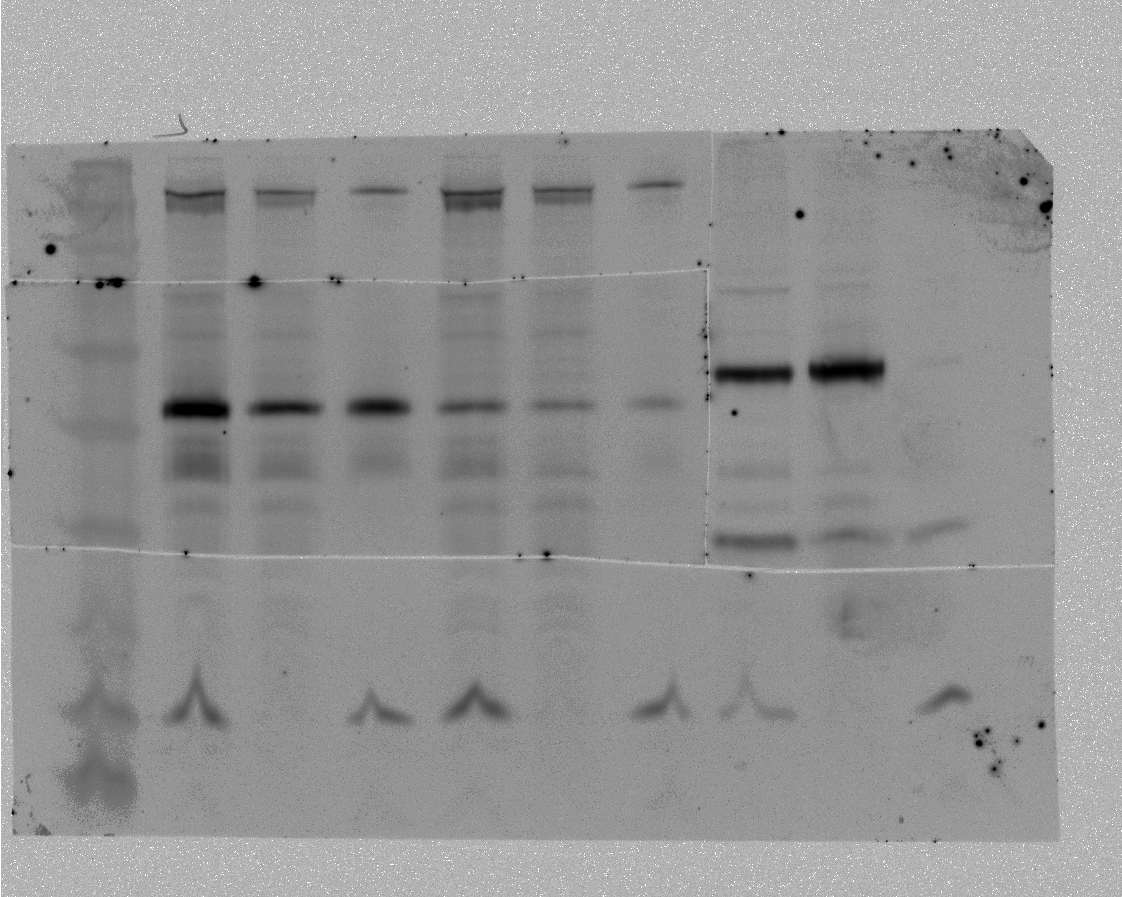

Supplement: Figure 1—figure supplement 1—source data 1. — Relevant bands for Figure 1—figure supplement 1C are highlighted with a red rectangle. L=ladder, x=lanes not used in Figure 1—figure supplement 1C. [file elife-83810-fig1-figsupp1-data1.zip › Figure 1-figure supplement 1-source data 1/Figure 1-figure supplement 1-source data 1 TBP H3.3 raw.tif]

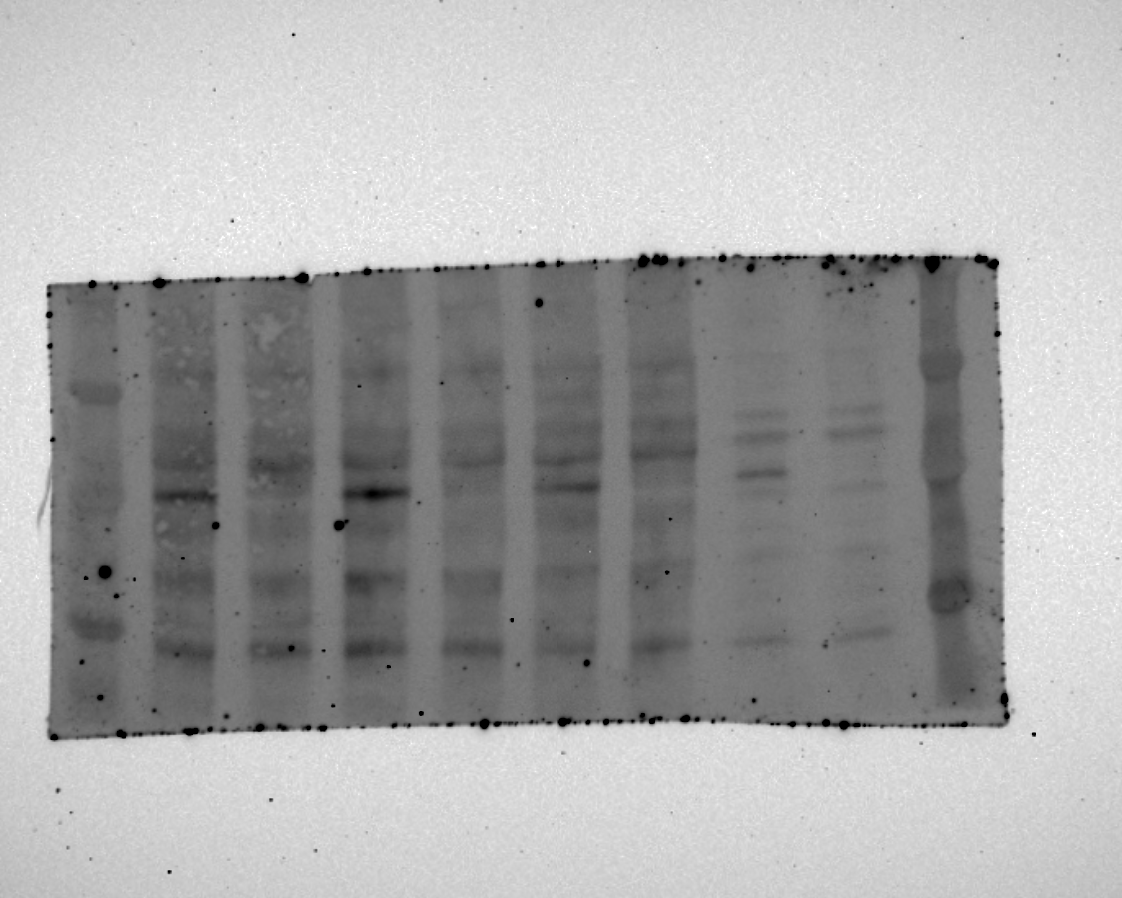

Supplement: Figure 2—figure supplement 1—source data 1. — Relevant bands for Figure 2—figure supplement 1A are highlighted with a red rectangle. L=ladder, x=lanes not used in Figure 2—figure supplement 1A. [file elife-83810-fig2-figsupp1-data1.zip › Figure 2-figure supplement 1-source data 1/Figure 2-figure supplement 1-source data 1 RPC7 raw.tif]

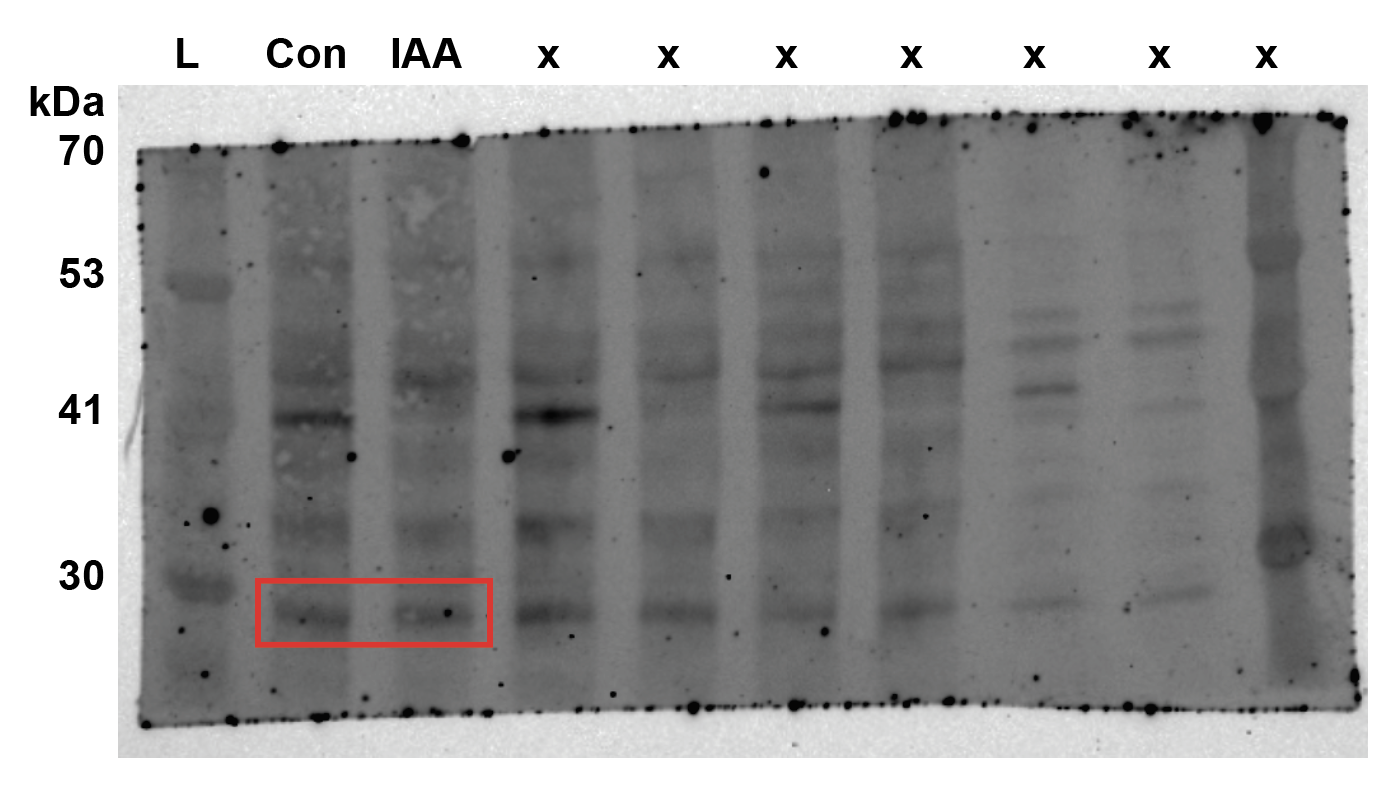

Supplement: Figure 2—figure supplement 1—source data 1. — Relevant bands for Figure 2—figure supplement 1A are highlighted with a red rectangle. L=ladder, x=lanes not used in Figure 2—figure supplement 1A. [file elife-83810-fig2-figsupp1-data1.zip › Figure 2-figure supplement 1-source data 1/Figure 2-figure supplement 1-source data 1 RPC7 annotated.tiff]

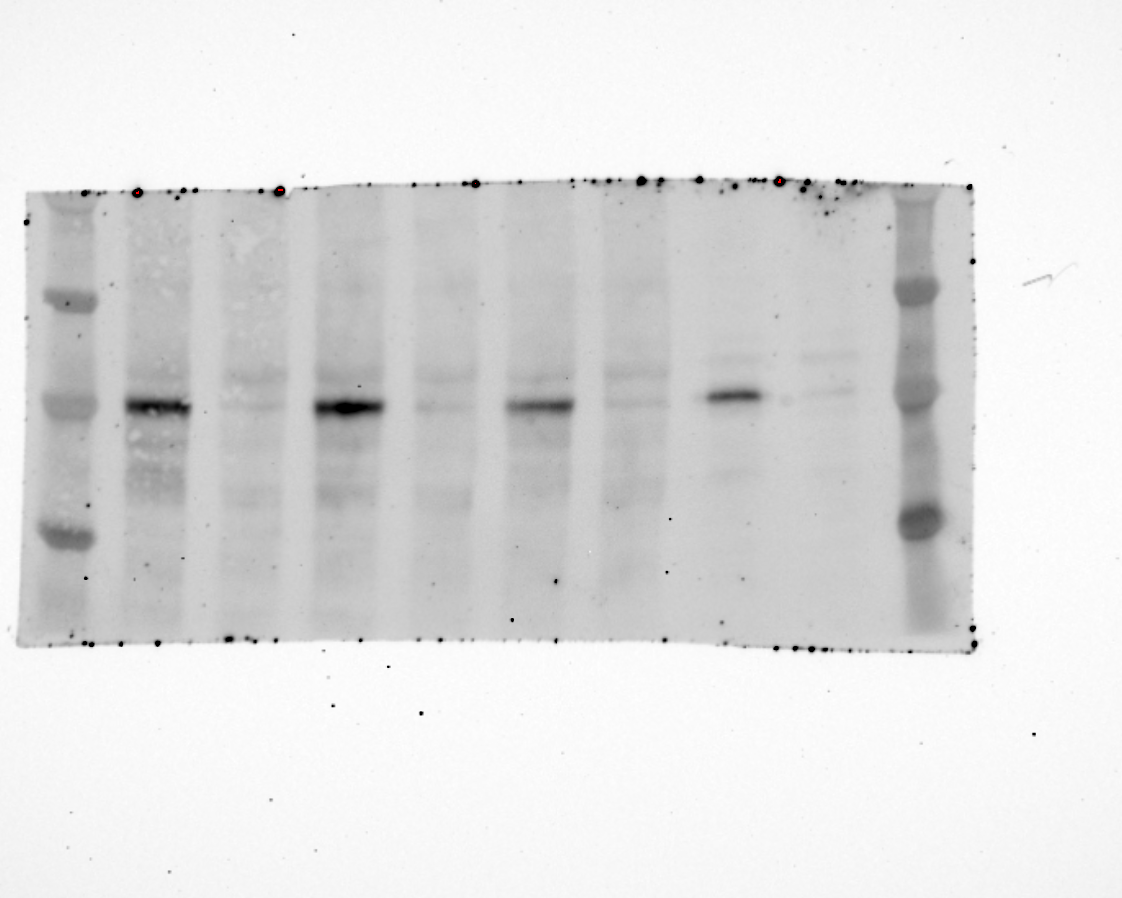

Supplement: Figure 2—figure supplement 1—source data 2. — Relevant bands for Figure 2—figure supplement 1A are highlighted with a red rectangle. L=ladder, x=lanes not used in Figure 2—figure supplement 1A. [file elife-83810-fig2-figsupp1-data2.zip › Figure 2-figure supplement 1-source data 2/Figure 2-figure supplement 1-source data 2 TBP raw.tif]

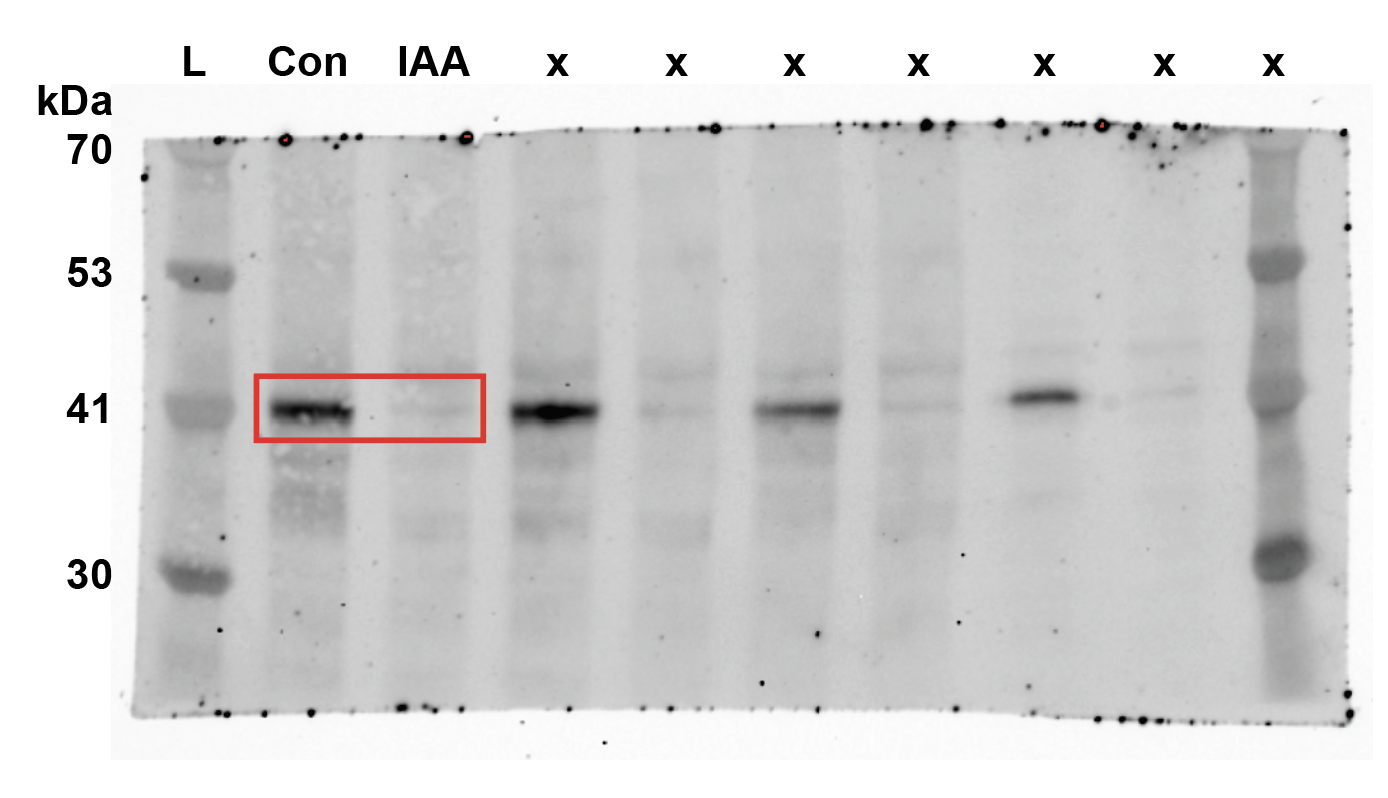

Supplement: Figure 2—figure supplement 1—source data 2. — Relevant bands for Figure 2—figure supplement 1A are highlighted with a red rectangle. L=ladder, x=lanes not used in Figure 2—figure supplement 1A. [file elife-83810-fig2-figsupp1-data2.zip › Figure 2-figure supplement 1-source data 2/Figure 2-figure supplement 1-source data 2 TBP annotated.tiff]

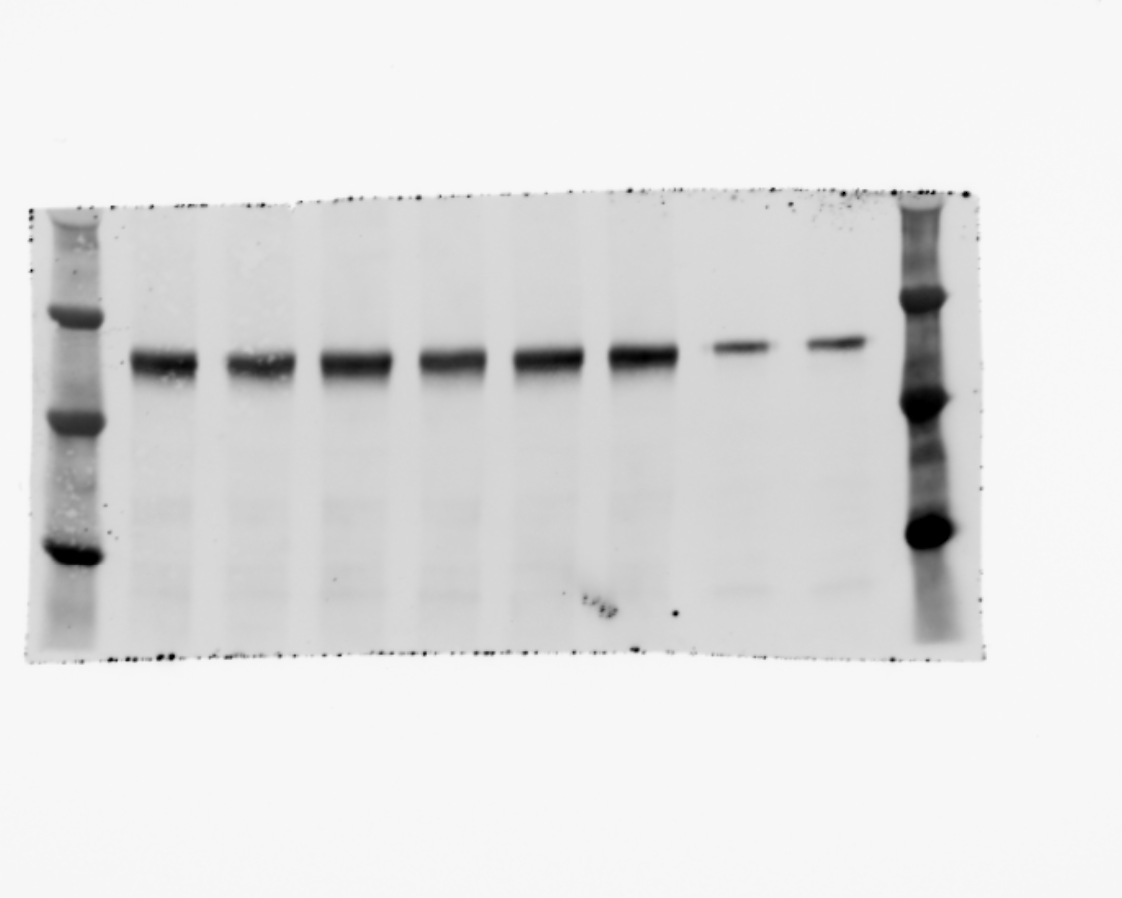

Supplement: Figure 2—figure supplement 1—source data 3. — Relevant bands for Figure 2—figure supplement 1A are highlighted with a red rectangle. L=ladder, x=lanes not used in Figure 2—figure supplement 1A. [file elife-83810-fig2-figsupp1-data3.zip › Figure 2-figure supplement 1-source data 3/Figure 2-figure supplement 1-source data 3 Tubulin raw.tif]

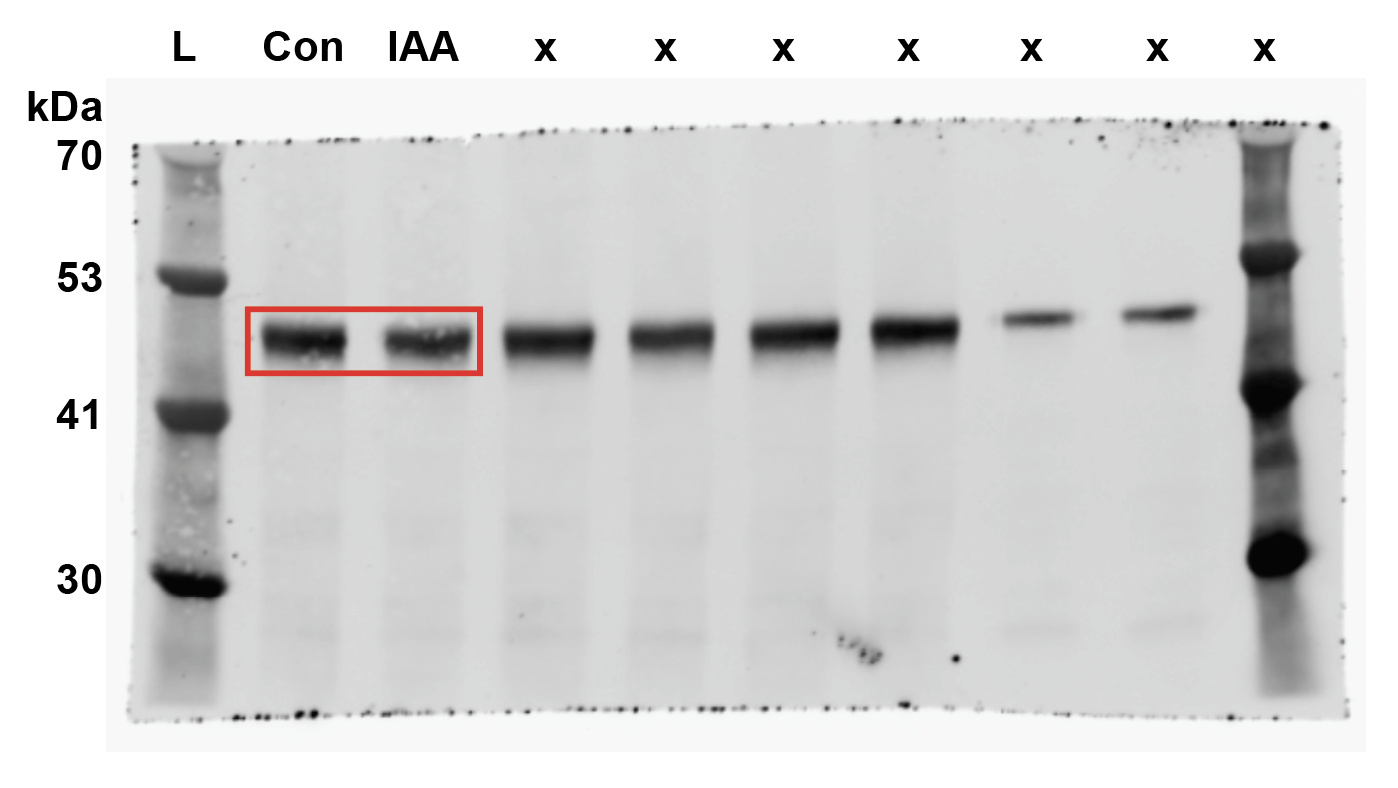

Supplement: Figure 2—figure supplement 1—source data 3. — Relevant bands for Figure 2—figure supplement 1A are highlighted with a red rectangle. L=ladder, x=lanes not used in Figure 2—figure supplement 1A. [file elife-83810-fig2-figsupp1-data3.zip › Figure 2-figure supplement 1-source data 3/Figure 2-figure supplement 1-source data 3 Tubulin annotated.tiff]

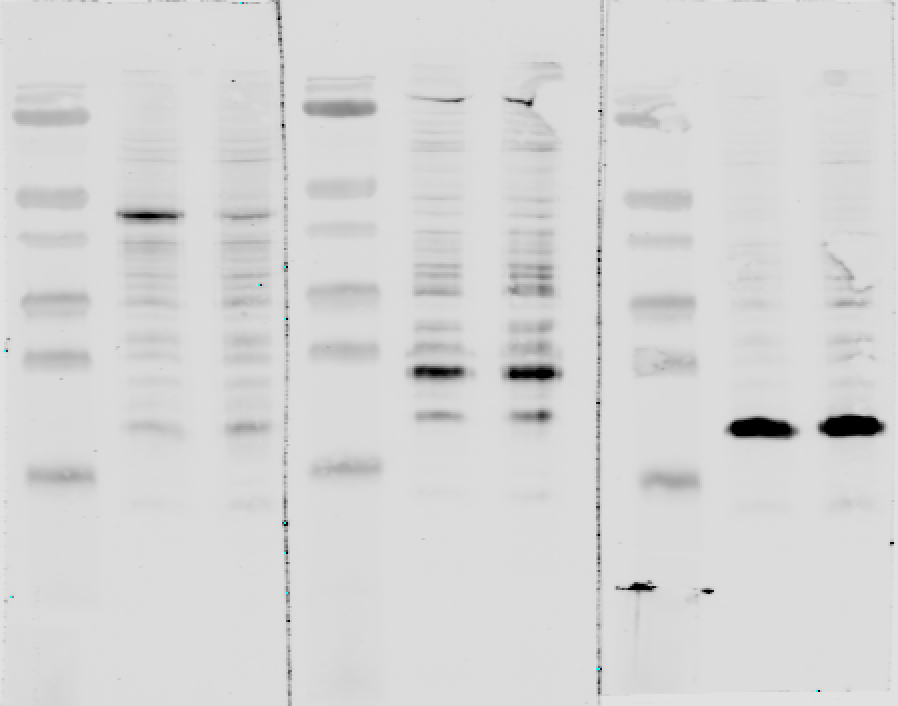

Supplement: Figure 4—source data 1. — Relevant bands for Figure 4A are highlighted with a red rectangle. L=ladder, x=lanes not used in Figure 4A. [file elife-83810-fig4-data1.zip › Figure 4-source data 1/Figure 4-source data 1 TRF2 H3K27me3 raw.tiff]

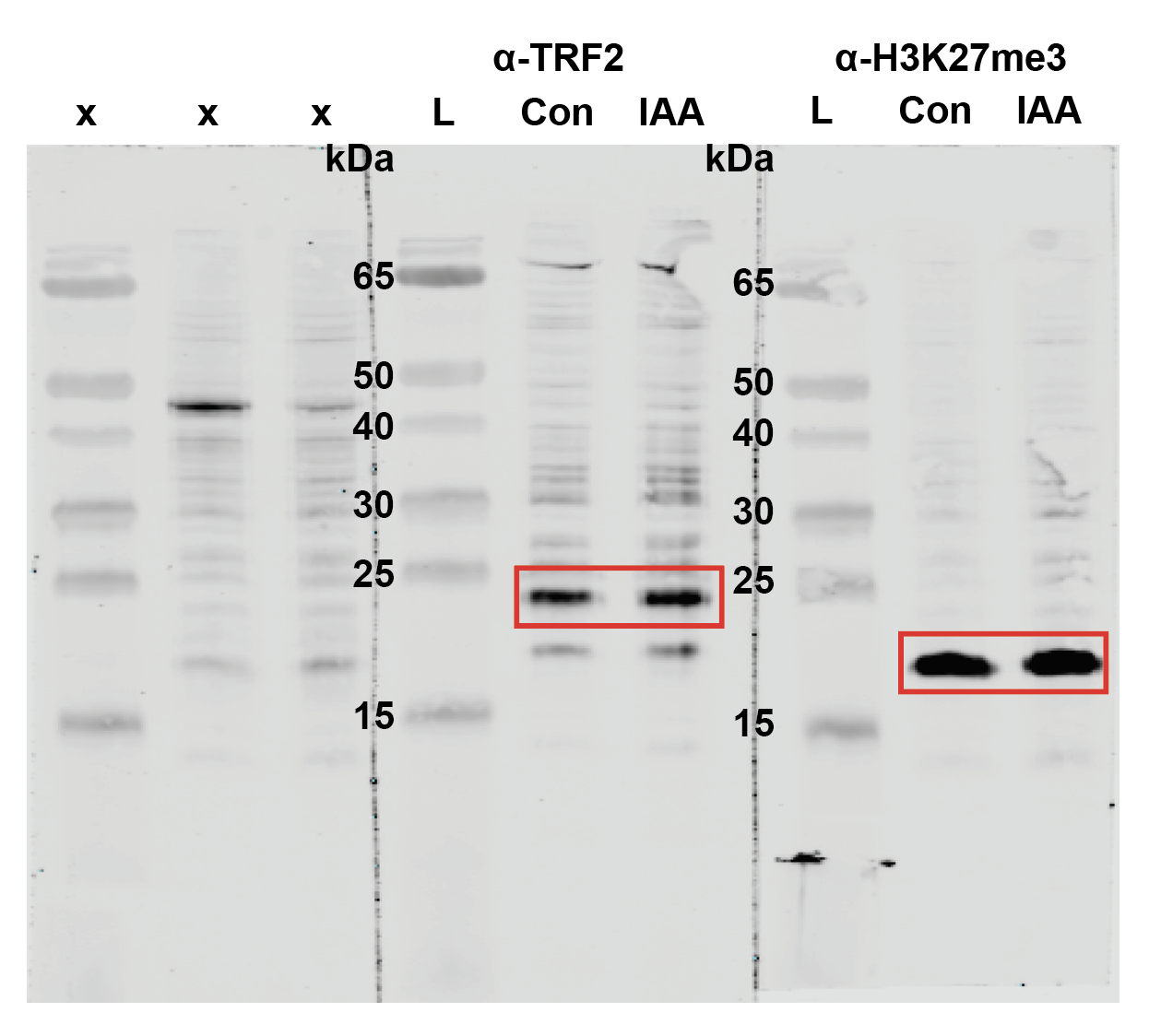

Supplement: Figure 4—source data 1. — Relevant bands for Figure 4A are highlighted with a red rectangle. L=ladder, x=lanes not used in Figure 4A. [file elife-83810-fig4-data1.zip › Figure 4-source data 1/Figure 4-source data 1 TRF2 H3K27me3 annotated.tiff]

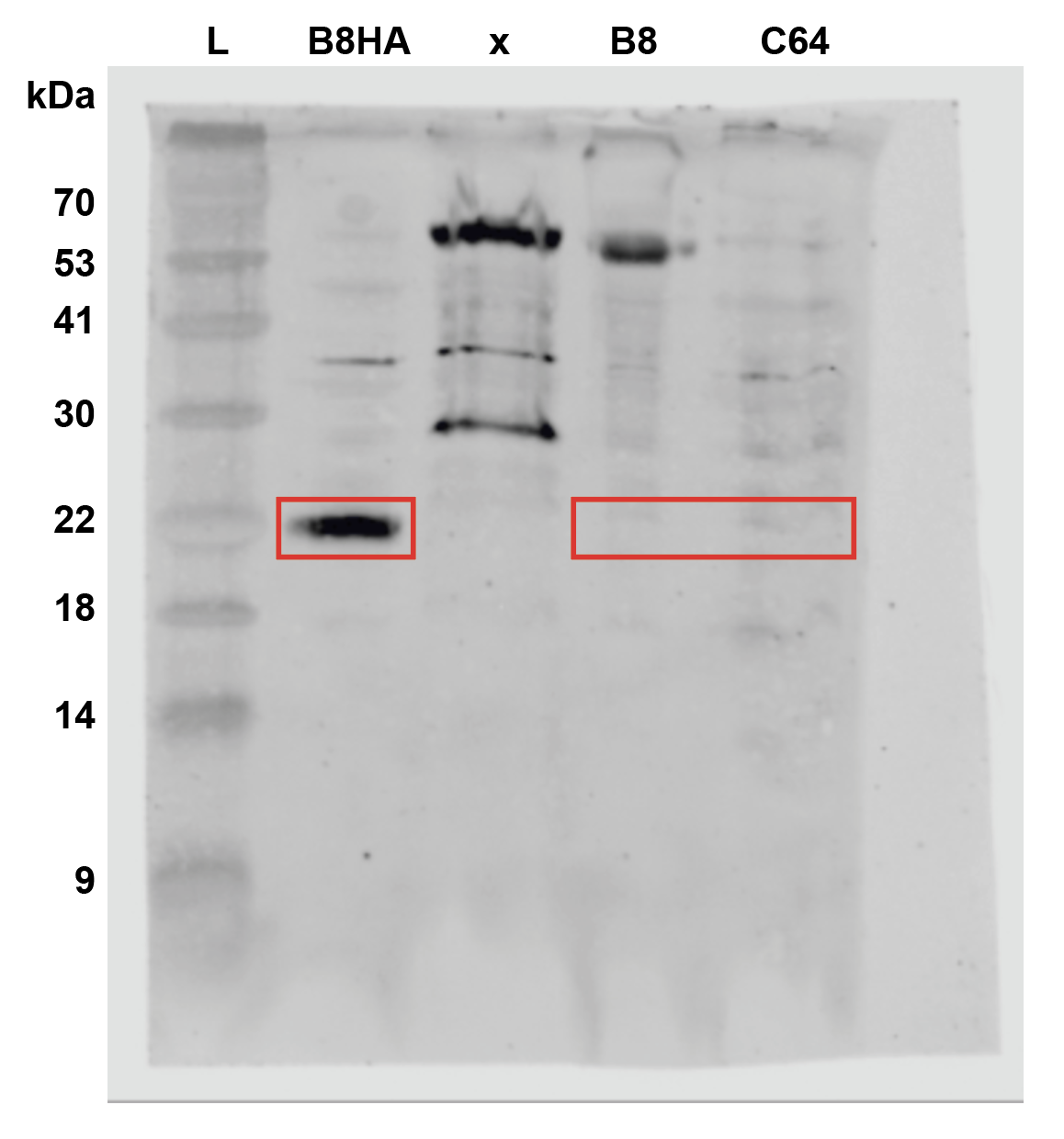

Supplement: Figure 4—source data 2. — Relevant bands for Figure 4B are highlighted with a red rectangle. L=ladder, x=lanes not used in Figure 4B. [file elife-83810-fig4-data2.zip › Figure 4-source data 2/Figure 4-source data 2 HA annotated.tiff]

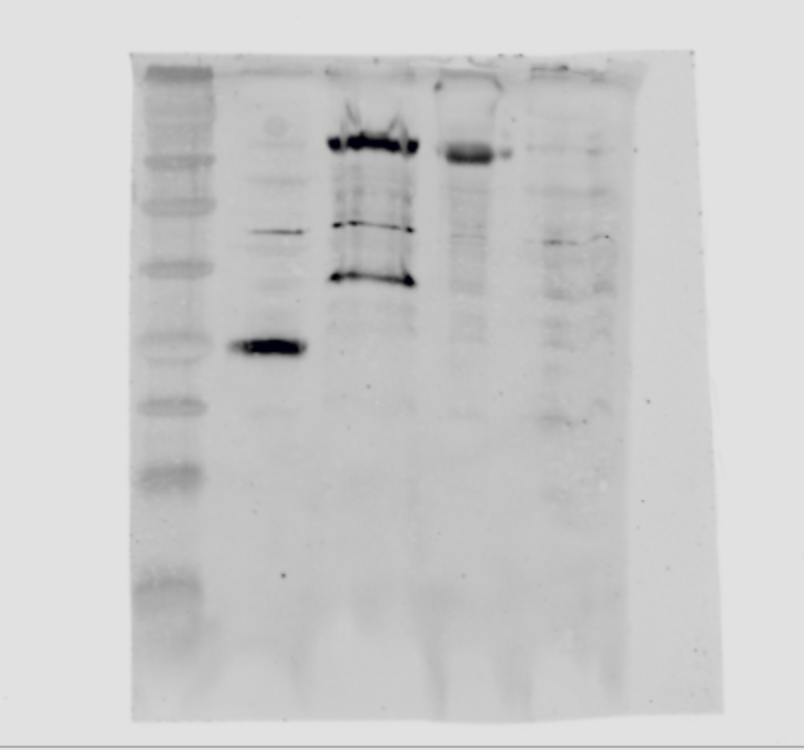

Supplement: Figure 4—source data 2. — Relevant bands for Figure 4B are highlighted with a red rectangle. L=ladder, x=lanes not used in Figure 4B. [file elife-83810-fig4-data2.zip › Figure 4-source data 2/Figure 4-source data 2 HA raw.tiff]

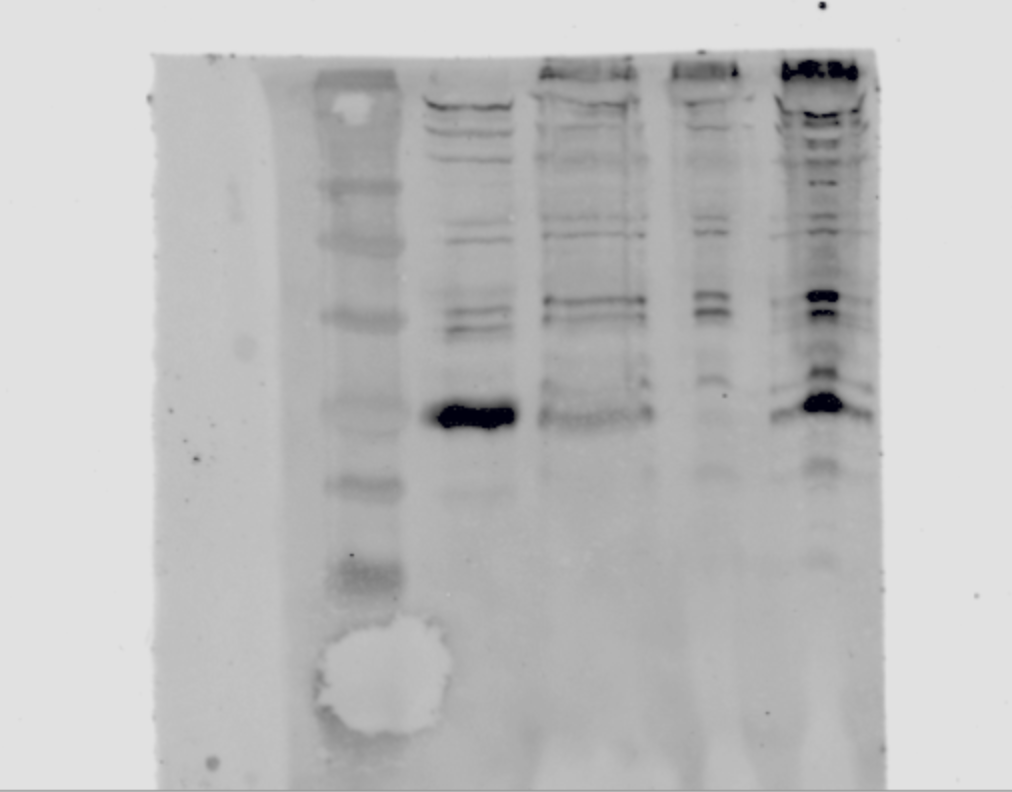

Supplement: Figure 4—source data 3. — Relevant bands for Figure 4B are highlighted with a red rectangle. L=ladder, x=lanes not used in Figure 4B. [file elife-83810-fig4-data3.zip › Figure 4-source data 3/Figure 4-source data 3 TRF2 raw.tiff]

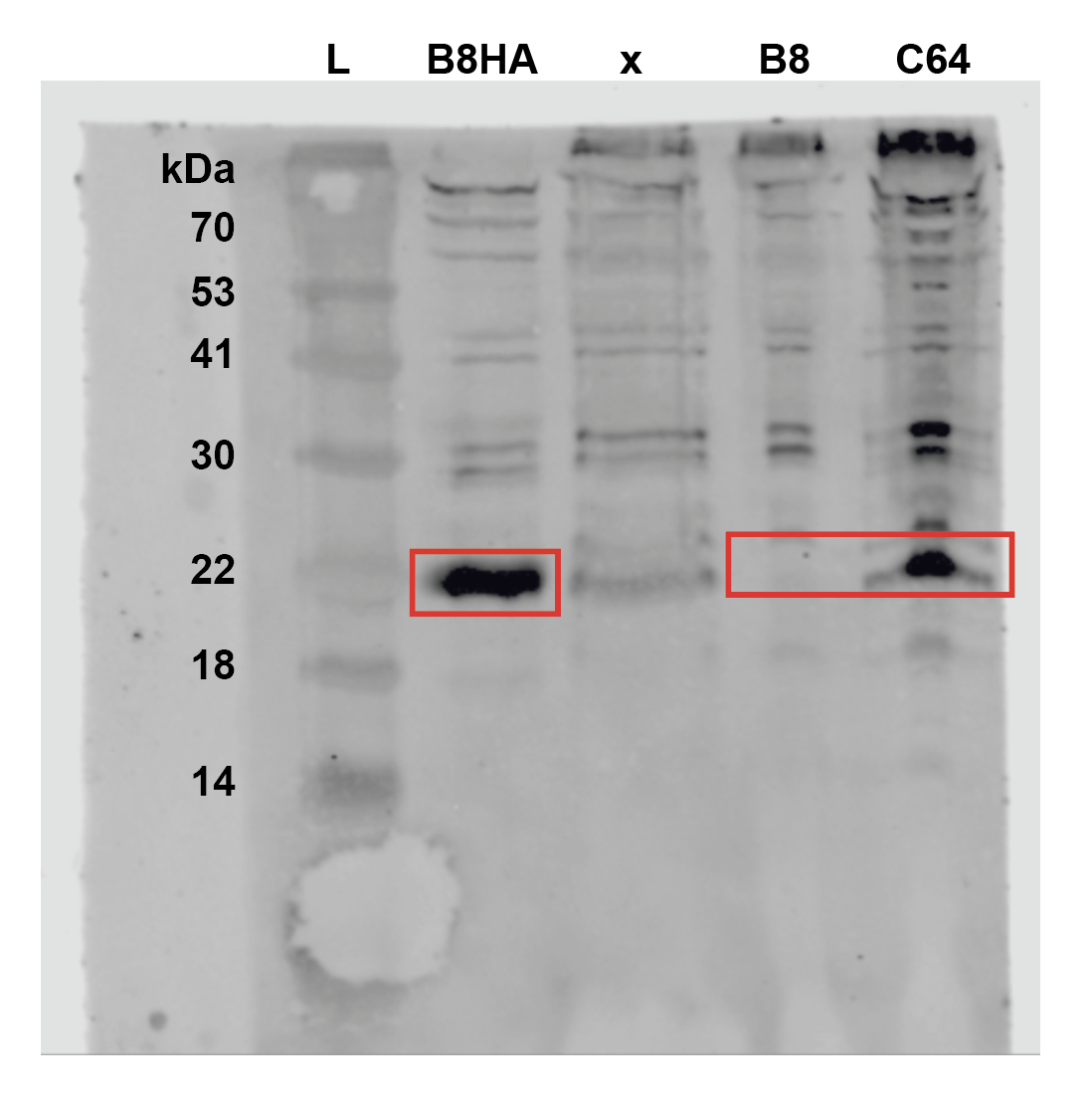

Supplement: Figure 4—source data 3. — Relevant bands for Figure 4B are highlighted with a red rectangle. L=ladder, x=lanes not used in Figure 4B. [file elife-83810-fig4-data3.zip › Figure 4-source data 3/Figure 4-source data 3 TRF2 annotated.tiff]

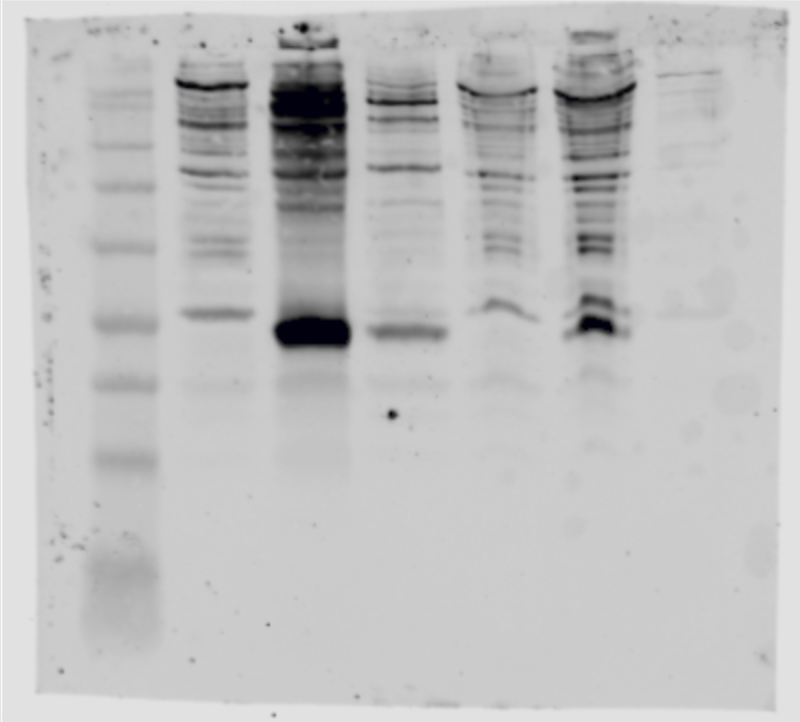

Supplement: Figure 4—figure supplement 1—source data 1. — Relevant bands for Figure 4—figure supplement 1F are highlighted with a red rectangle. L=ladder, x=lanes not used in Figure 4—figure supplement 1F. [file elife-83810-fig4-figsupp1-data1.zip › Figure 4-figure supplement 1-source data 1/Figure 4-figure supplement 1-source data 1 TRF2 raw.tiff]

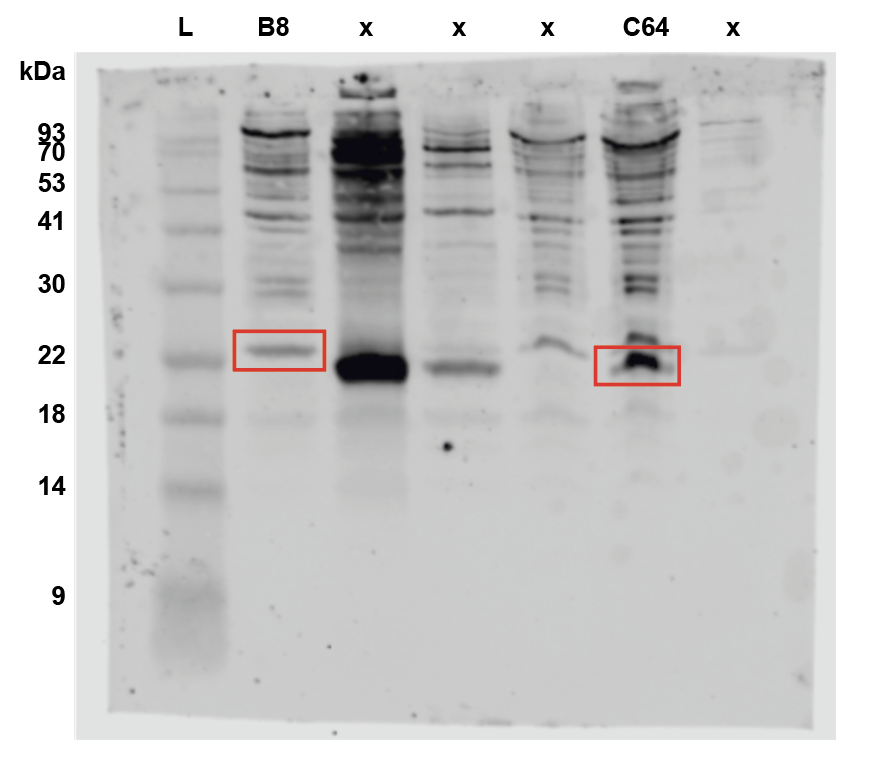

Supplement: Figure 4—figure supplement 1—source data 1. — Relevant bands for Figure 4—figure supplement 1F are highlighted with a red rectangle. L=ladder, x=lanes not used in Figure 4—figure supplement 1F. [file elife-83810-fig4-figsupp1-data1.zip › Figure 4-figure supplement 1-source data 1/Figure 4-figure supplement 1-source data 1 TRF2 annotated.tiff]

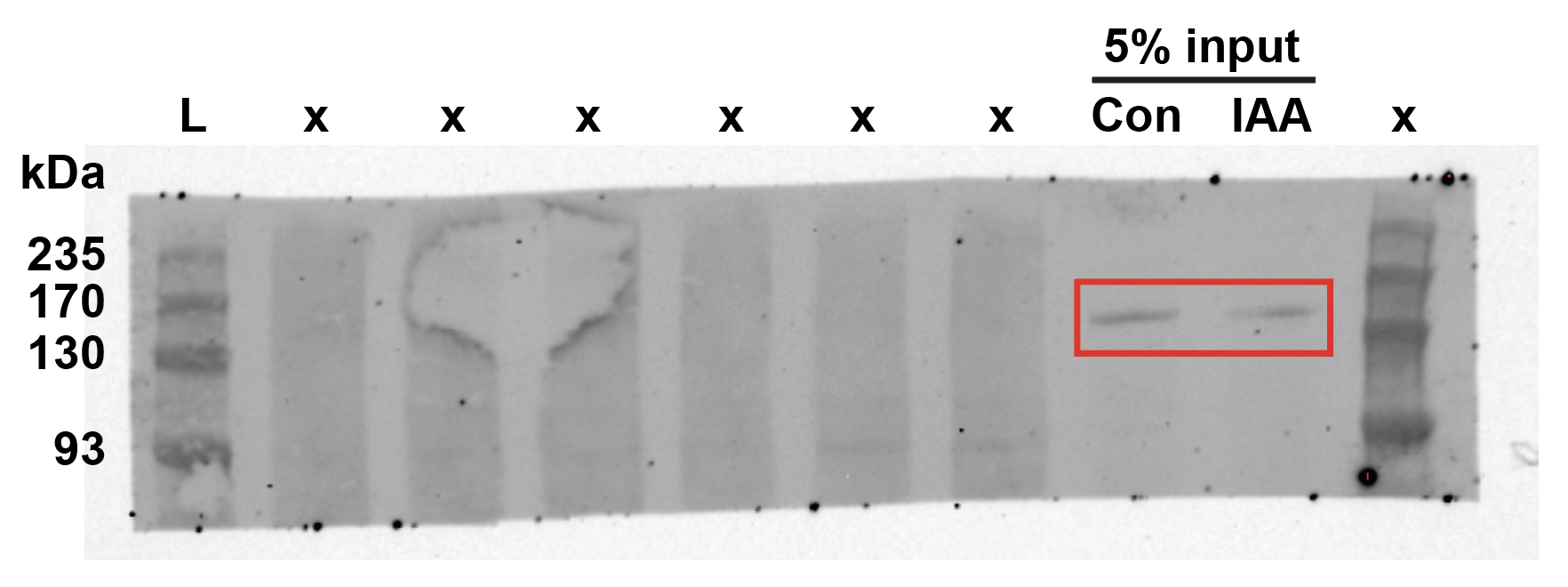

Supplement: Figure 5—source data 1. — Input represents 5% of the protein samples used in the TAF4 IPs. Relevant bands for Figure 5A are highlighted with a red rectangle. L=ladder, x=lanes not used in analyses. Relevant bands for Figure 5A are highlighted with a red rectangle. L=ladder, x=lanes not used in Figure 5A. [file elife-83810-fig5-data1.zip › Figure 5-source data 1/Figure 5-source data 1 TAF4 annotated.tiff]

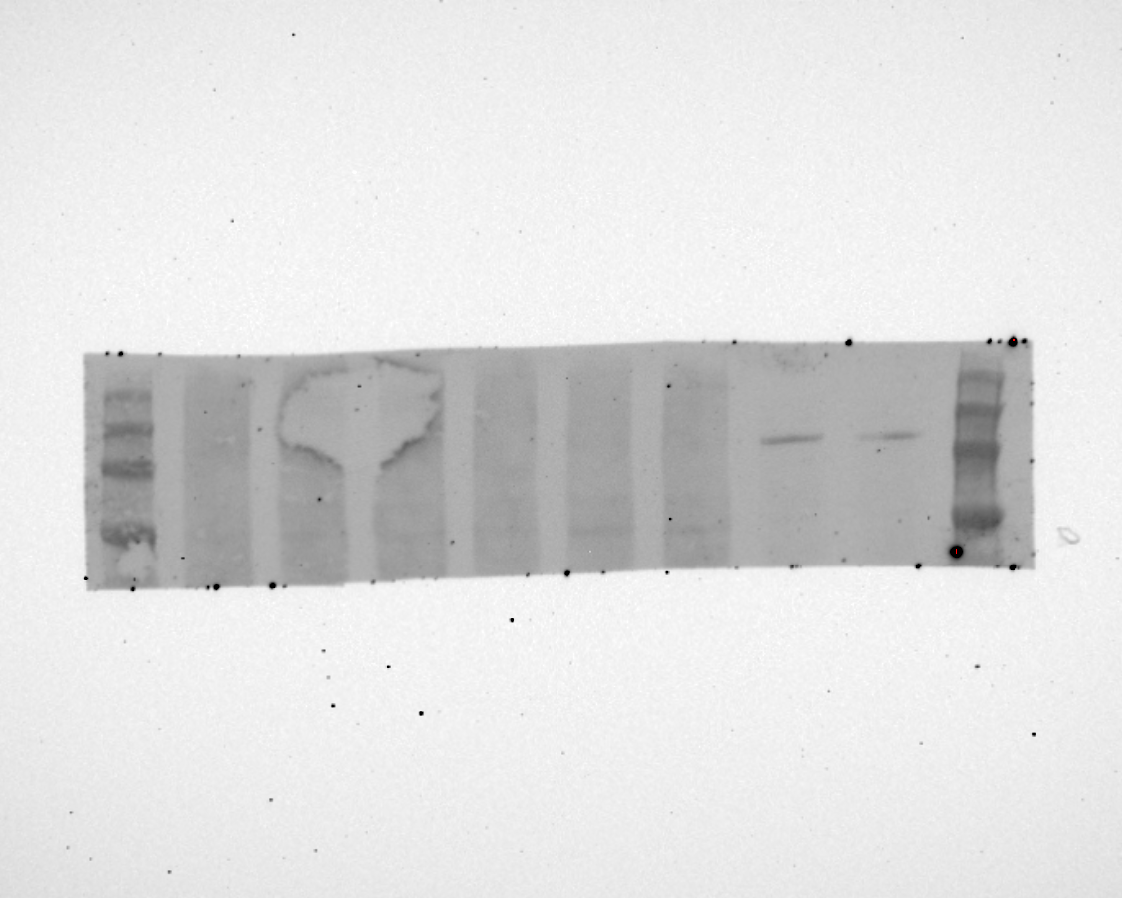

Supplement: Figure 5—source data 1. — Input represents 5% of the protein samples used in the TAF4 IPs. Relevant bands for Figure 5A are highlighted with a red rectangle. L=ladder, x=lanes not used in analyses. Relevant bands for Figure 5A are highlighted with a red rectangle. L=ladder, x=lanes not used in Figure 5A. [file elife-83810-fig5-data1.zip › Figure 5-source data 1/Figure 5-source data 1 TAF4 raw.tif]

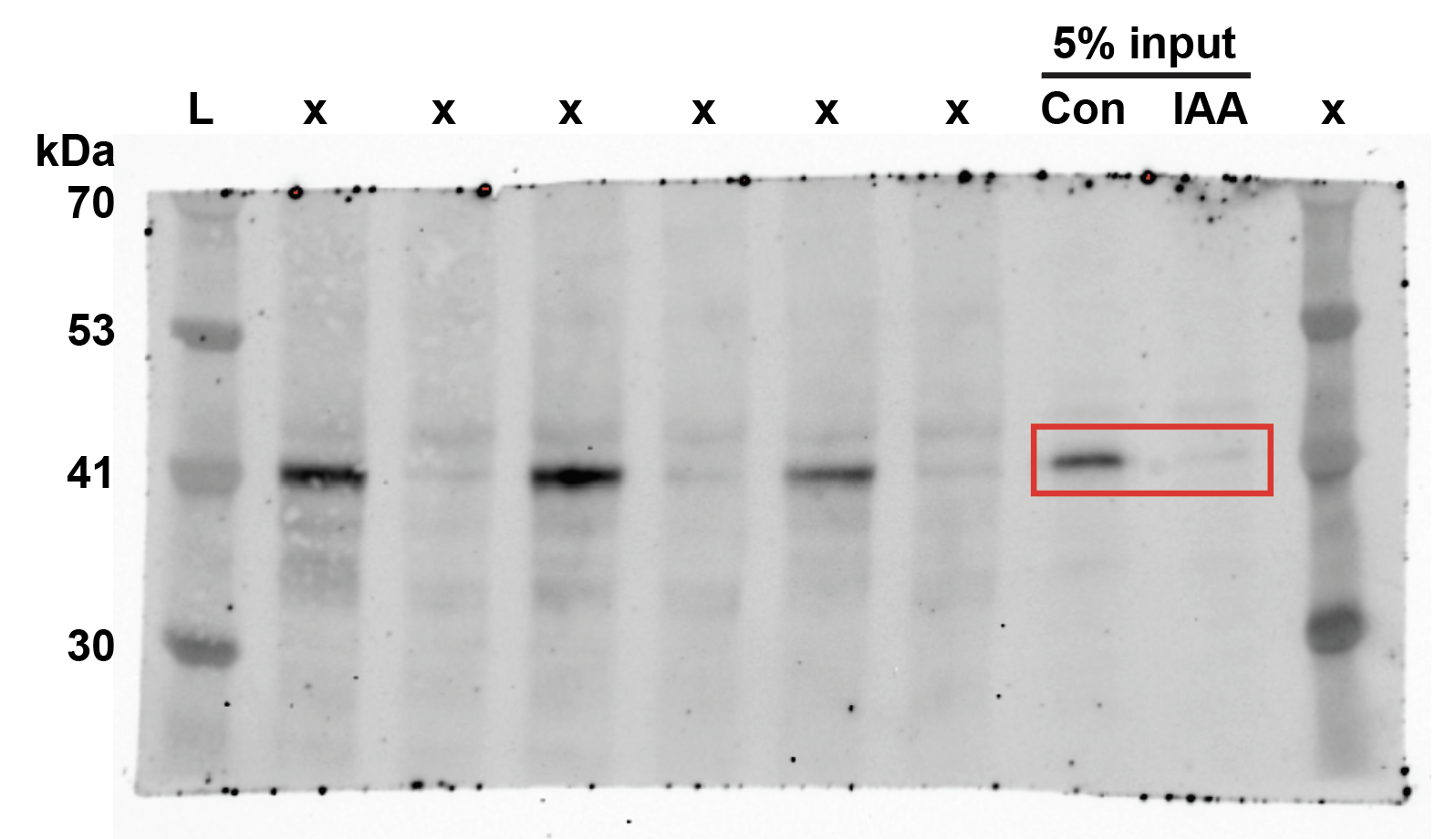

Supplement: Figure 5—source data 2. — Input represents 5% of the protein samples used in the TAF4 IPs. Relevant bands for Figure 5A are highlighted with a red rectangle. L=ladder, x=lanes not used in analyses. Relevant bands for Figure 5A are highlighted with a red rectangle. L=ladder, x=lanes not used in Figure 5A. [file elife-83810-fig5-data2.zip › Figure 5-source data 2/Figure 5-source data 2 TBP annotated.tiff]

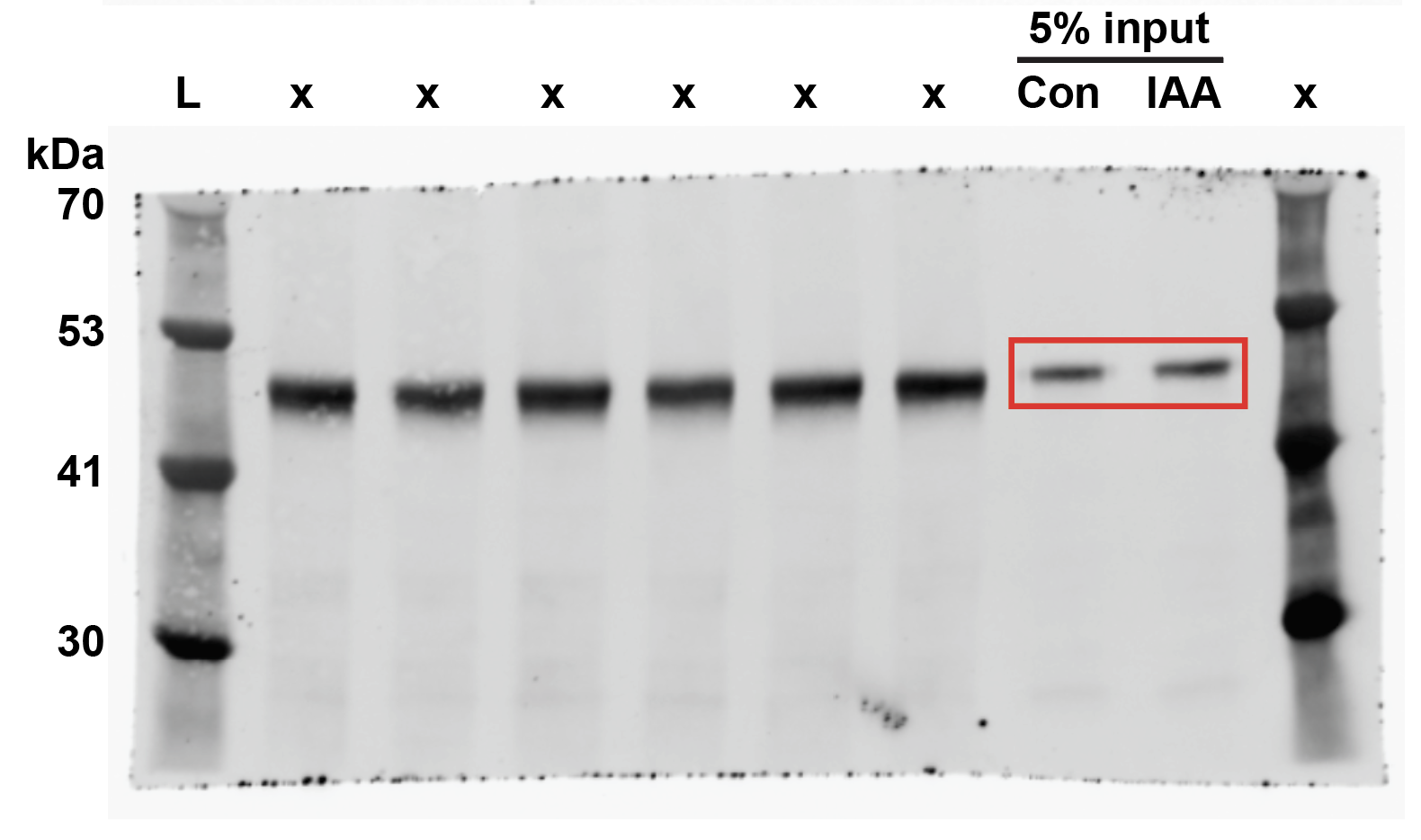

Supplement: Figure 5—source data 3. — Input represents 5% of the protein samples used in the TAF4 IPs. Relevant bands for Figure 5A are highlighted with a red rectangle. L=ladder, x=lanes not used in analyses. Relevant bands for Figure 5A are highlighted with a red rectangle. L=ladder, x=lanes not used in Figure 5A. [file elife-83810-fig5-data3.zip › Figure 5-source data 3/Figure 5-source data 3 Tubulin annotated.tiff]

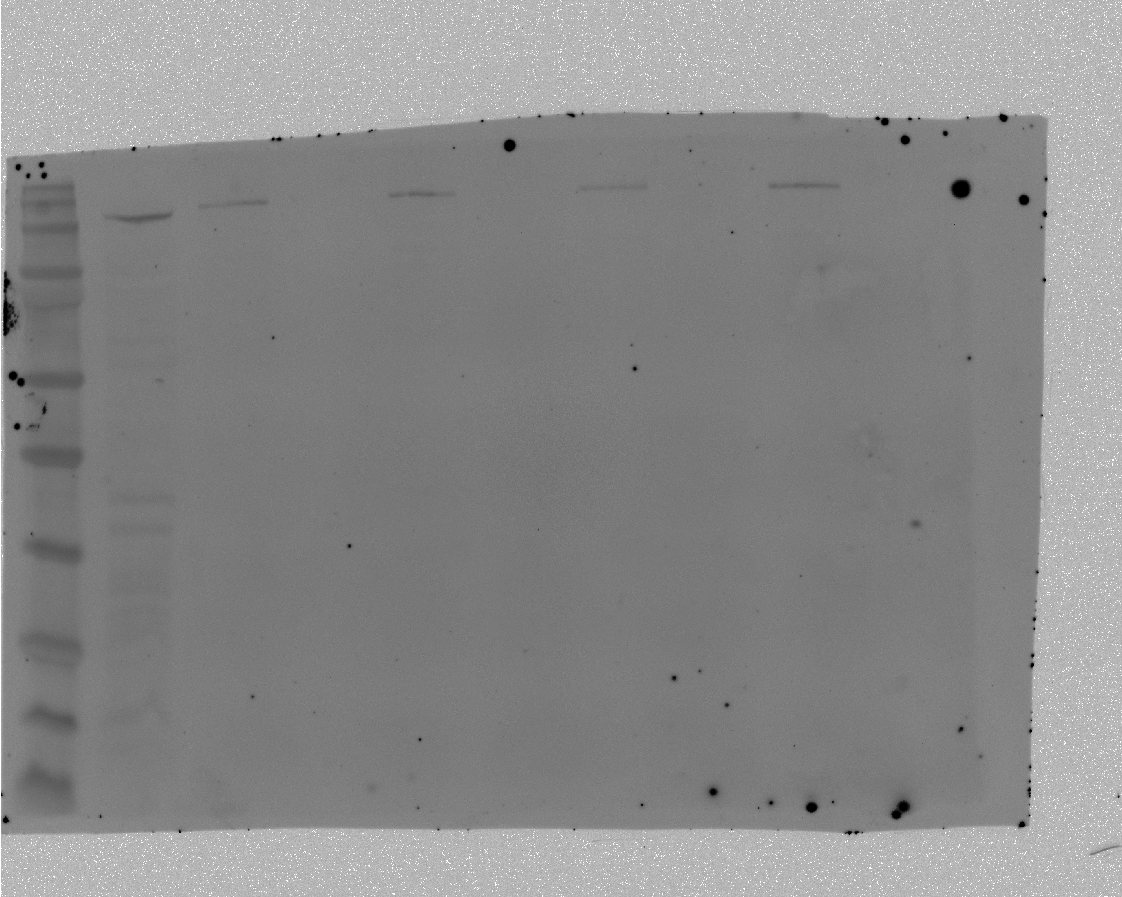

Supplement: Figure 5—source data 4. — Relevant bands for Figure 5A are highlighted with a red rectangle. L=ladder, x=lanes not used in Figure 5A. [file elife-83810-fig5-data4.zip › Figure 5-source data 4/Figure 5-source data 4 TAF4 raw.tif]

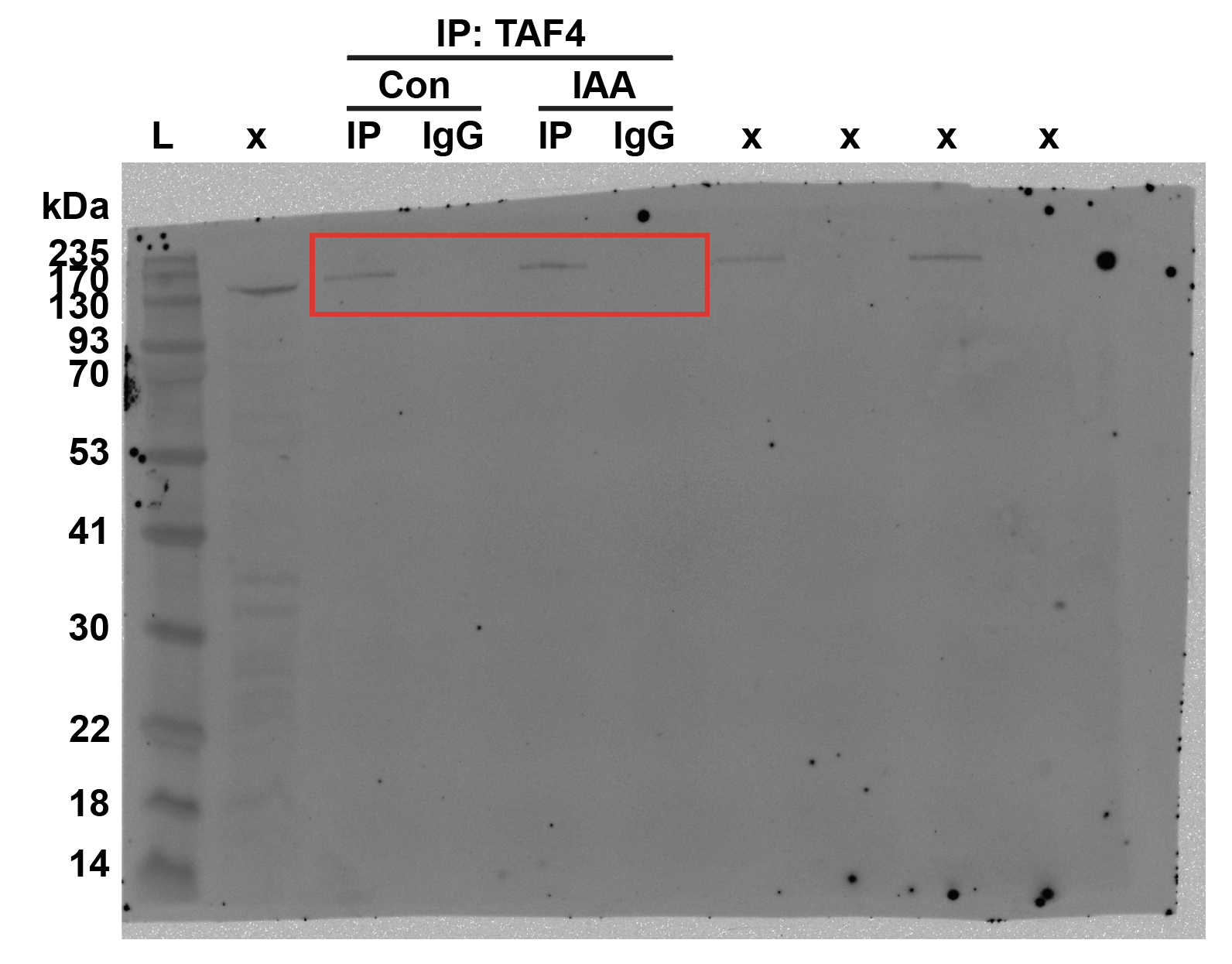

Supplement: Figure 5—source data 4. — Relevant bands for Figure 5A are highlighted with a red rectangle. L=ladder, x=lanes not used in Figure 5A. [file elife-83810-fig5-data4.zip › Figure 5-source data 4/Figure 5-source data 4 TAF4 annotated.tiff]

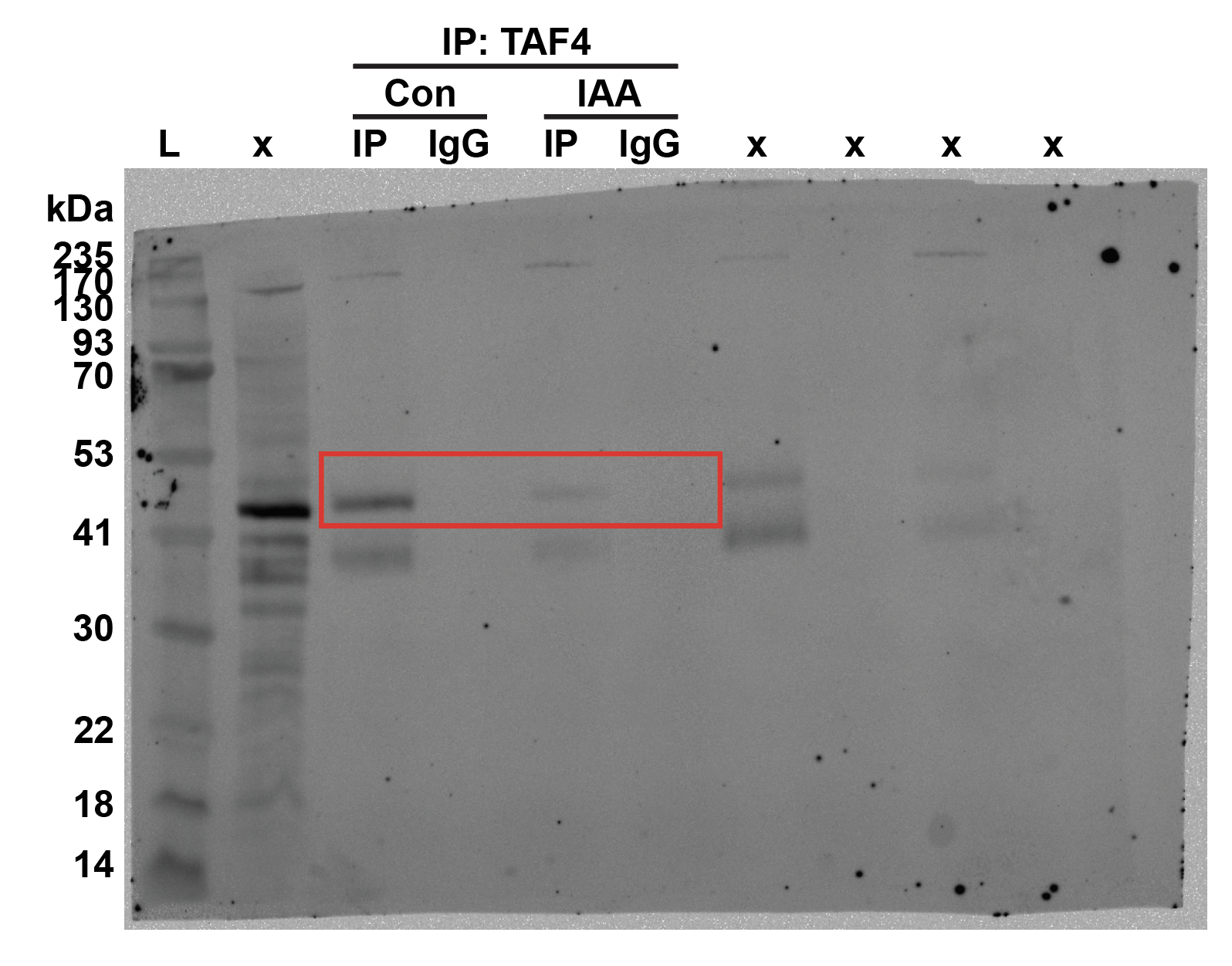

Supplement: Figure 5—source data 5. — Relevant bands for Figure 5A are highlighted with a red rectangle. L=ladder, x=lanes not used in Figure 5A. [file elife-83810-fig5-data5.zip › Figure 5-source data 5/Figure 5-source data 5 TBP annotated.tiff]

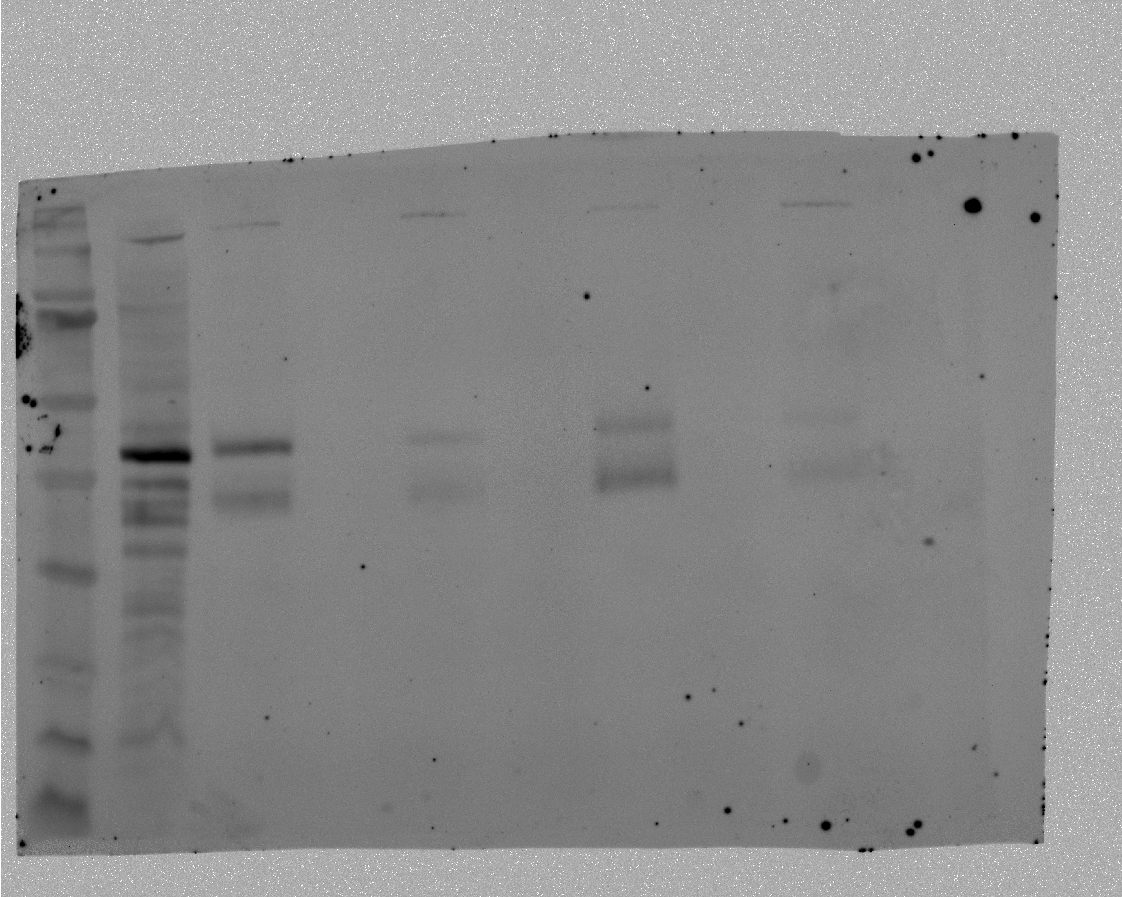

Supplement: Figure 5—source data 5. — Relevant bands for Figure 5A are highlighted with a red rectangle. L=ladder, x=lanes not used in Figure 5A. [file elife-83810-fig5-data5.zip › Figure 5-source data 5/Figure 5-source data 5 TBP raw.tif]

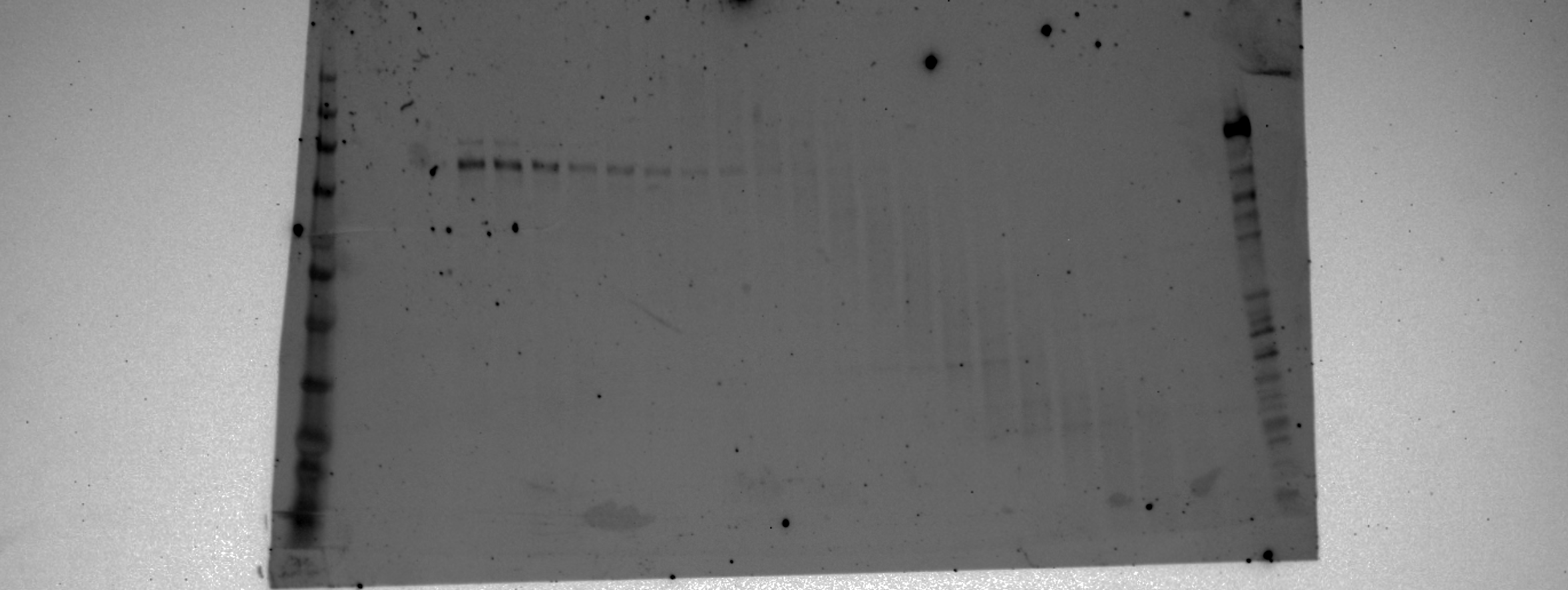

Supplement: Figure 5—figure supplement 1—source data 1. — Relevant bands for Figure 5—figure supplement 1C are highlighted with a red rectangle. L=ladder, in = input, x=lanes not used in Figure 5—figure supplement 1C. [file elife-83810-fig5-figsupp1-data1.zip › Figure 5-figure supplement 1-source data 1/Figure 5-figure supplement 1-source data 1 TAF4 raw.tif]

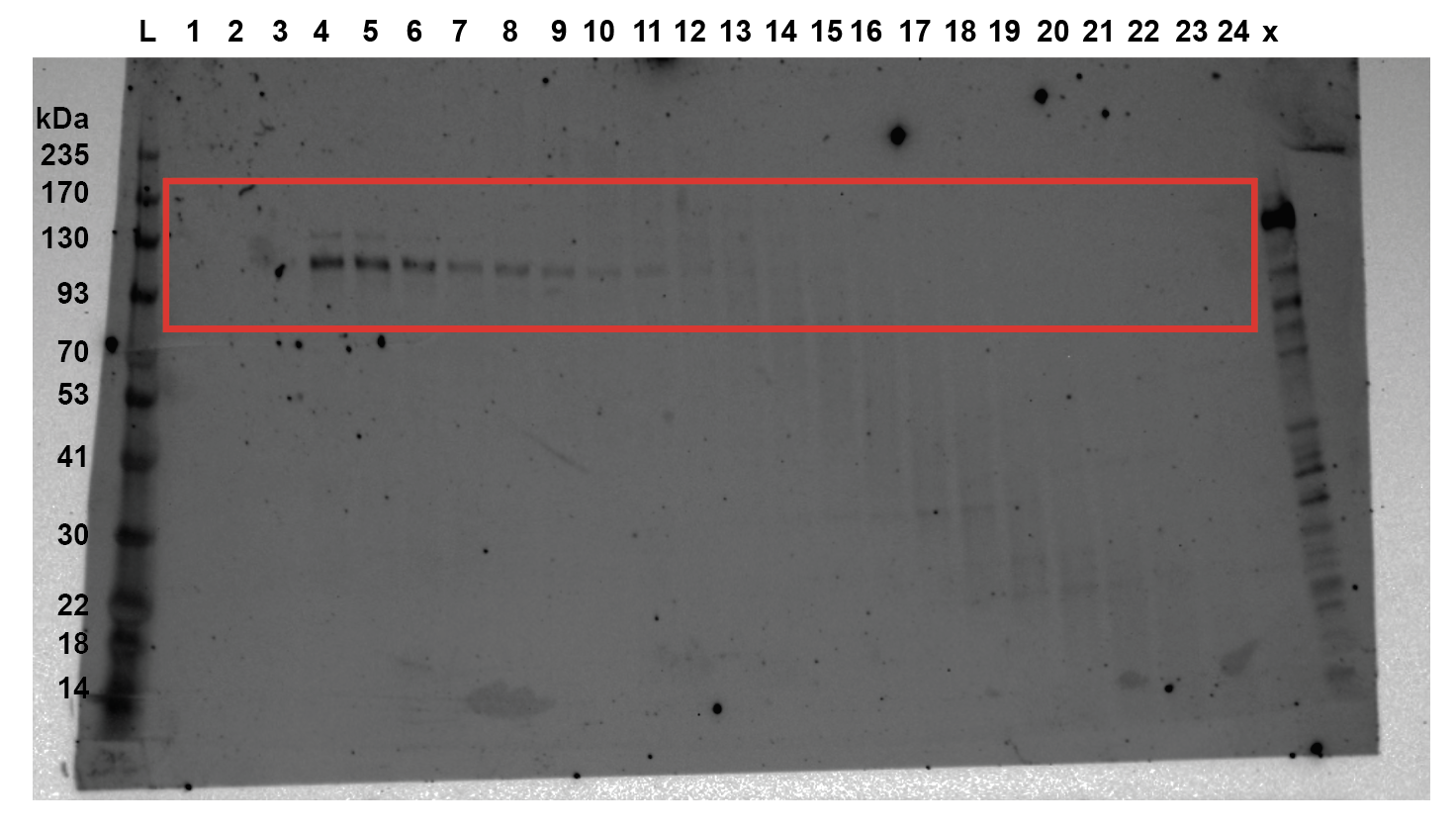

Supplement: Figure 5—figure supplement 1—source data 1. — Relevant bands for Figure 5—figure supplement 1C are highlighted with a red rectangle. L=ladder, in = input, x=lanes not used in Figure 5—figure supplement 1C. [file elife-83810-fig5-figsupp1-data1.zip › Figure 5-figure supplement 1-source data 1/Figure 5-figure supplement 1-source data 1 TAF4 annotated.tiff]

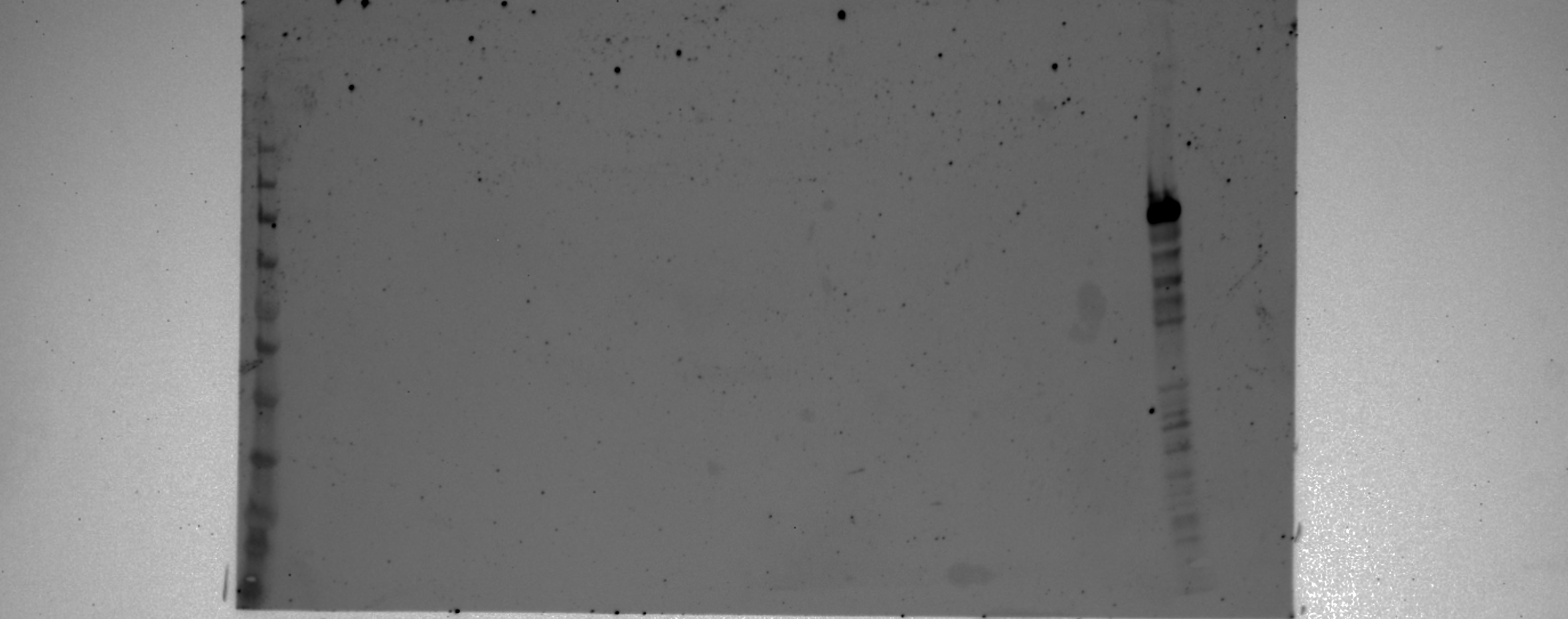

Supplement: Figure 5—figure supplement 1—source data 2. — Relevant bands for Figure 5—figure supplement 1C are highlighted with a red rectangle. L=ladder, in = input, x=lanes not used in Figure 5—figure supplement 1C. [file elife-83810-fig5-figsupp1-data2.zip › Figure 5-figure supplement 1-source data 2/Figure 5-figure supplement 1-source data 2 TAF4 raw.tif]

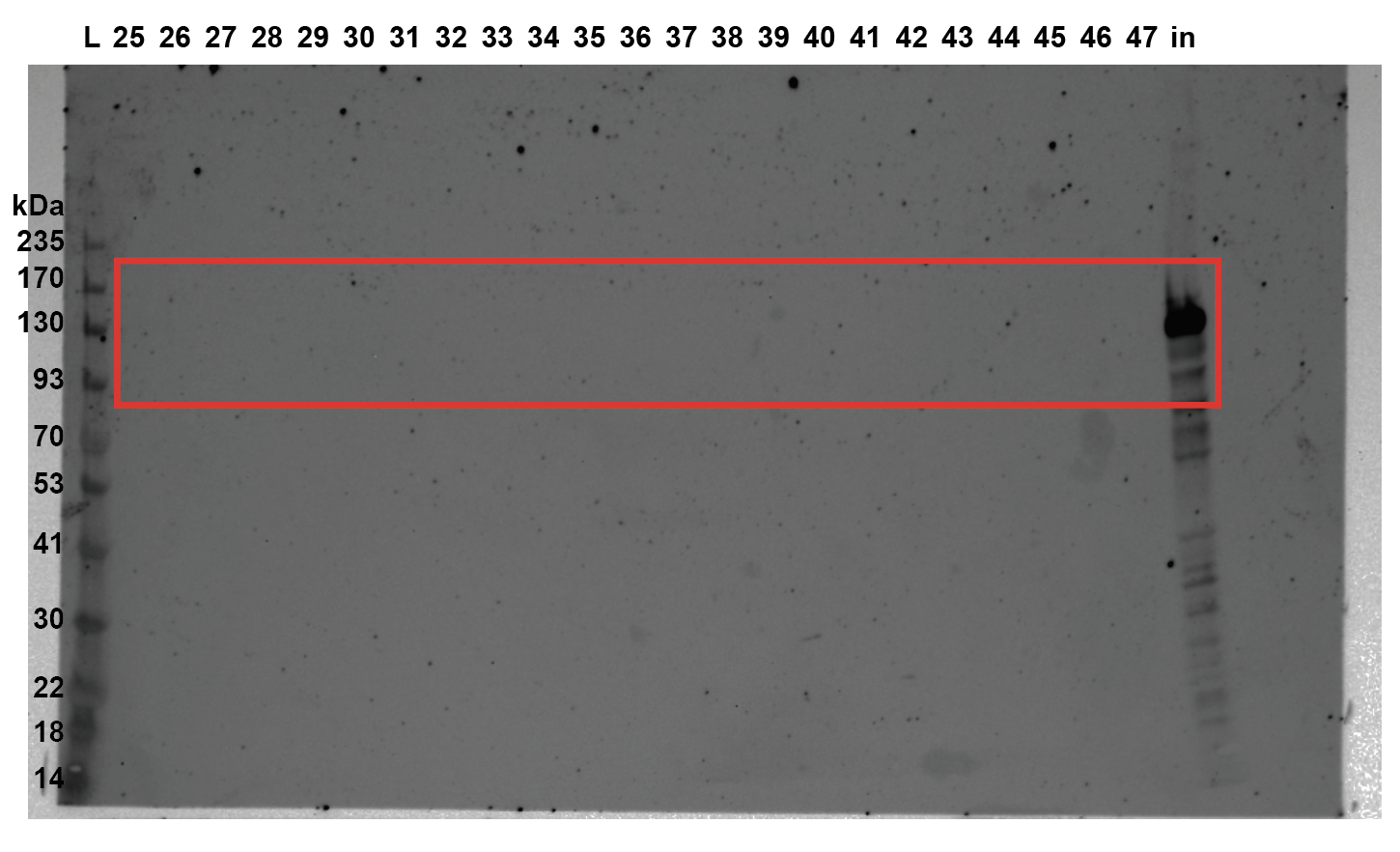

Supplement: Figure 5—figure supplement 1—source data 2. — Relevant bands for Figure 5—figure supplement 1C are highlighted with a red rectangle. L=ladder, in = input, x=lanes not used in Figure 5—figure supplement 1C. [file elife-83810-fig5-figsupp1-data2.zip › Figure 5-figure supplement 1-source data 2/Figure 5-figure supplement 1-source data 2 TAF4 annotated.tiff]

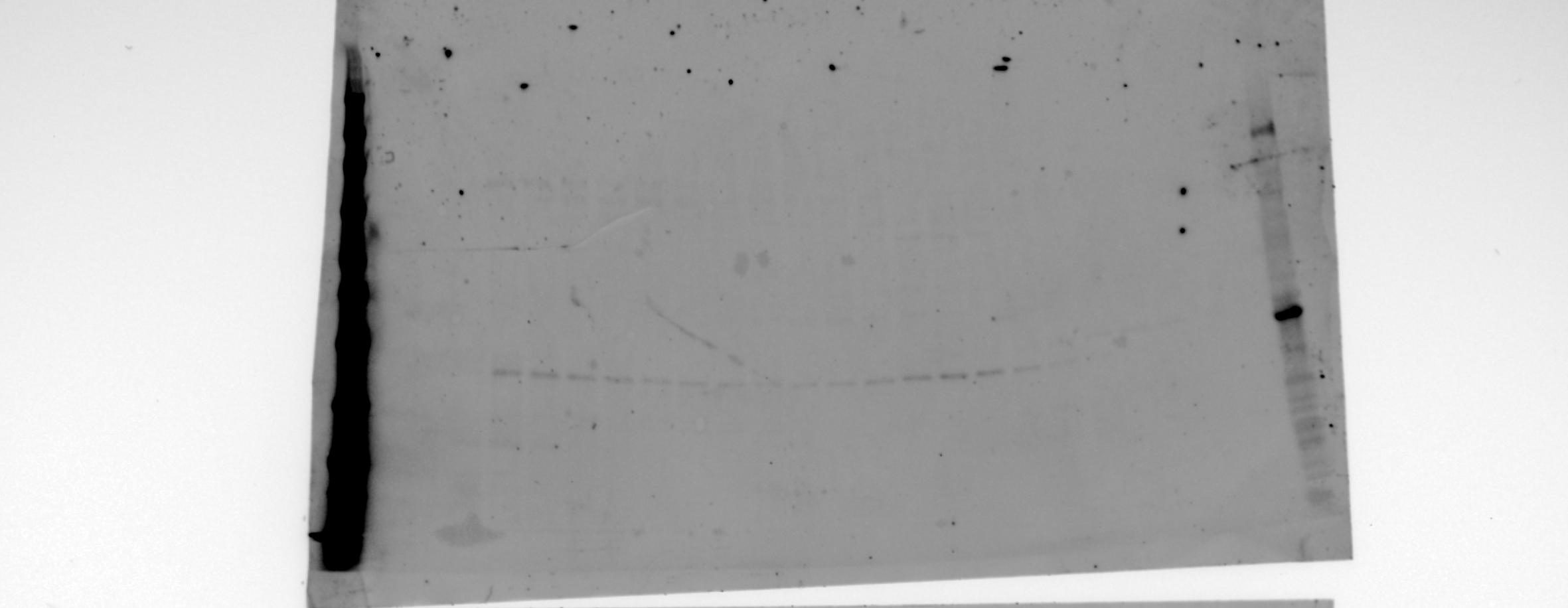

Supplement: Figure 5—figure supplement 1—source data 3. — Relevant bands for Figure 5—figure supplement 1C are highlighted with a red rectangle. L=ladder, in = input, x=lanes not used in Figure 5—figure supplement 1C. [file elife-83810-fig5-figsupp1-data3.zip › Figure 5-figure supplement 1-source data 3/Figure 5-figure supplement 1-source data 3 TBP raw.tif]

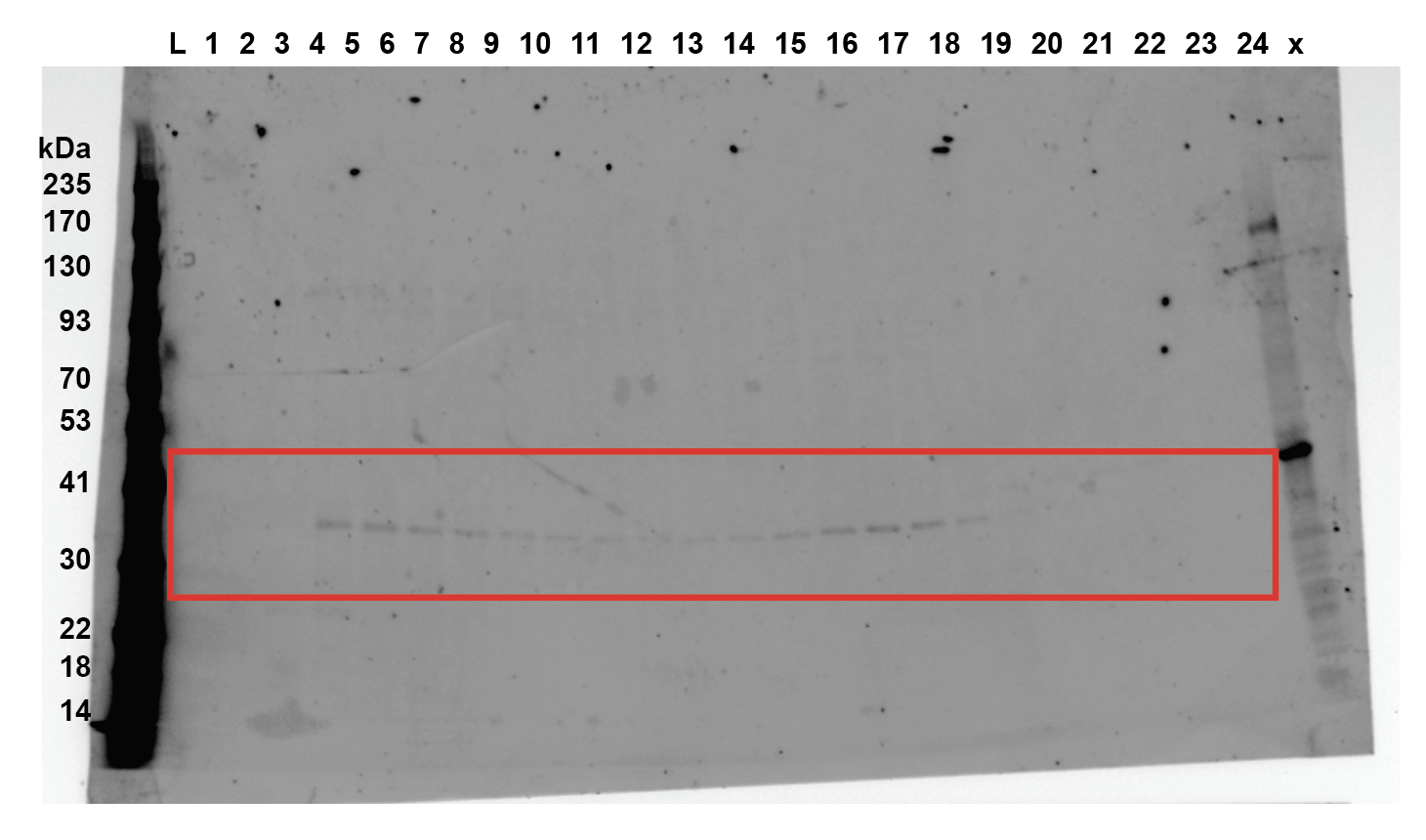

Supplement: Figure 5—figure supplement 1—source data 3. — Relevant bands for Figure 5—figure supplement 1C are highlighted with a red rectangle. L=ladder, in = input, x=lanes not used in Figure 5—figure supplement 1C. [file elife-83810-fig5-figsupp1-data3.zip › Figure 5-figure supplement 1-source data 3/Figure 5-figure supplement 1-source data 3 TBP annotated.tiff]

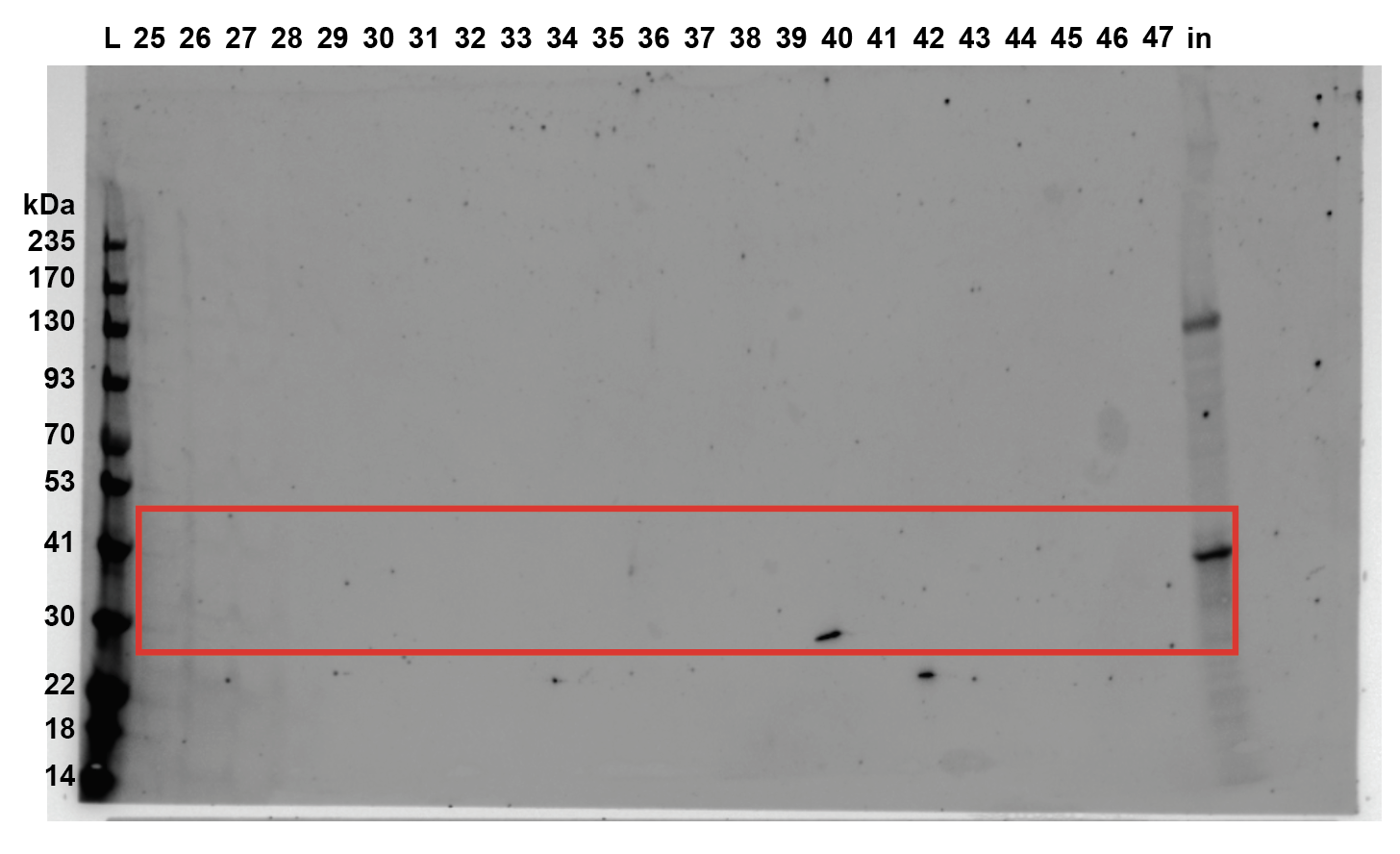

Supplement: Figure 5—figure supplement 1—source data 4. — Relevant bands for Figure 5—figure supplement 1C are highlighted with a red rectangle. L=ladder, in = input, x=lanes not used in Figure 5—figure supplement 1C. [file elife-83810-fig5-figsupp1-data4.zip › Figure 5-figure supplement 1-source data 4/Figure 5-figure supplement 1-source data 4 TBP annotated.tiff]

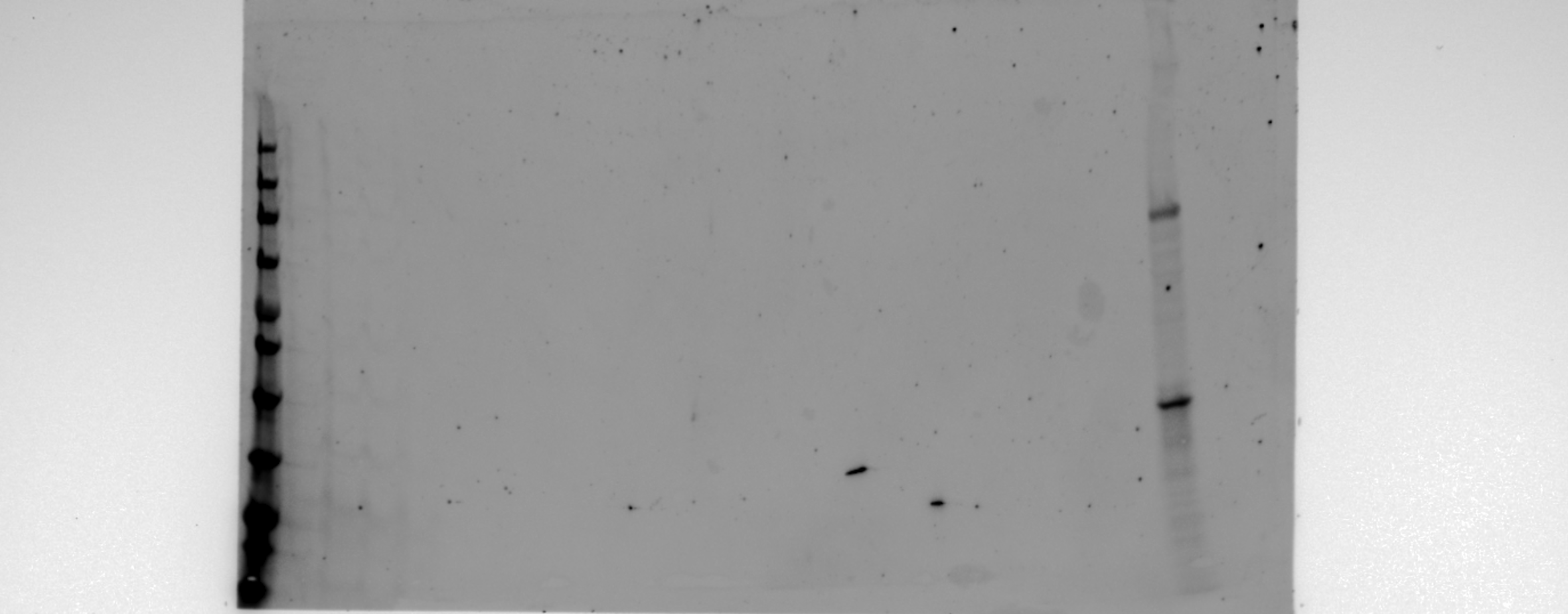

Supplement: Figure 5—figure supplement 1—source data 4. — Relevant bands for Figure 5—figure supplement 1C are highlighted with a red rectangle. L=ladder, in = input, x=lanes not used in Figure 5—figure supplement 1C. [file elife-83810-fig5-figsupp1-data4.zip › Figure 5-figure supplement 1-source data 4/Figure 5-figure supplement 1-source data 4 TBP raw.tif]

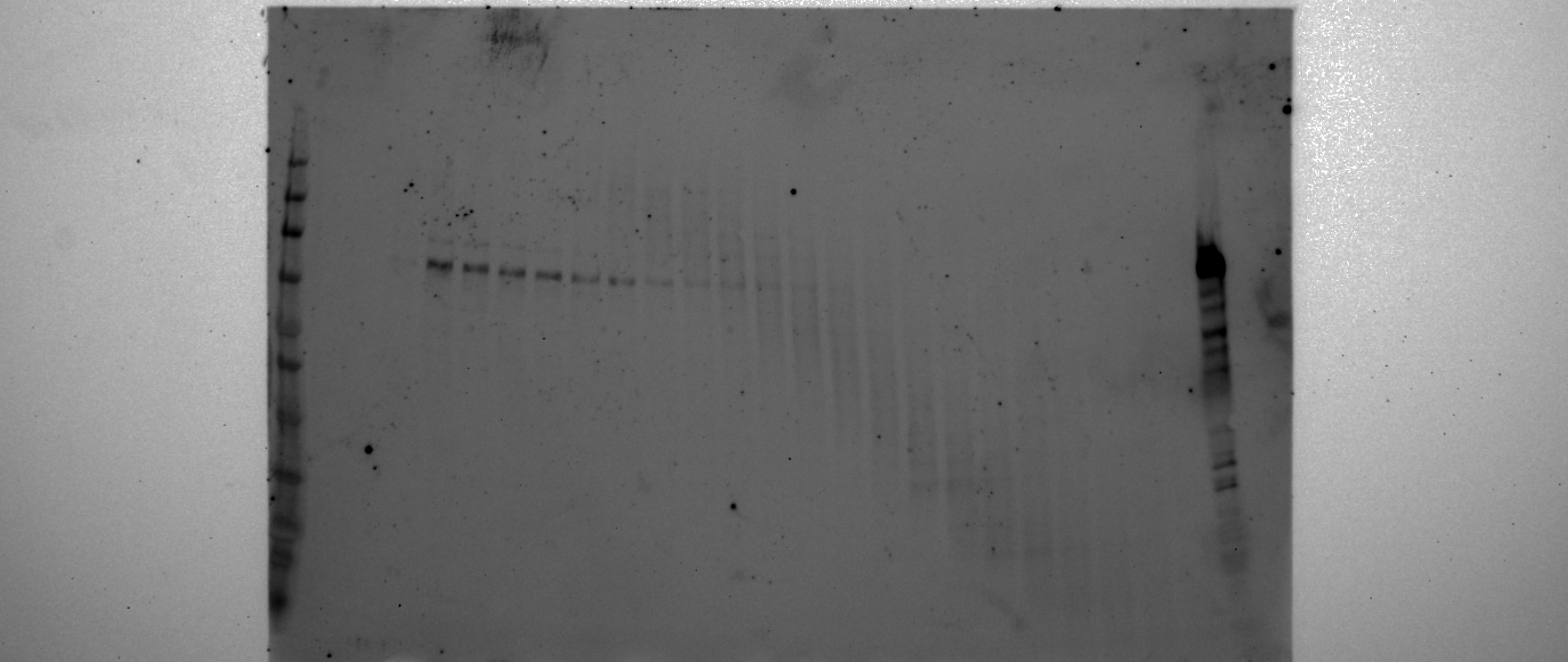

Supplement: Figure 5—figure supplement 1—source data 5. — Relevant bands for Figure 5—figure supplement 1C are highlighted with a red rectangle. L=ladder, in = input, x=lanes not used in Figure 5—figure supplement 1C. [file elife-83810-fig5-figsupp1-data5.zip › Figure 5-figure supplement 1-source data 5/Figure 5-figure supplement 1-source data 5 TAF4 raw.tif]

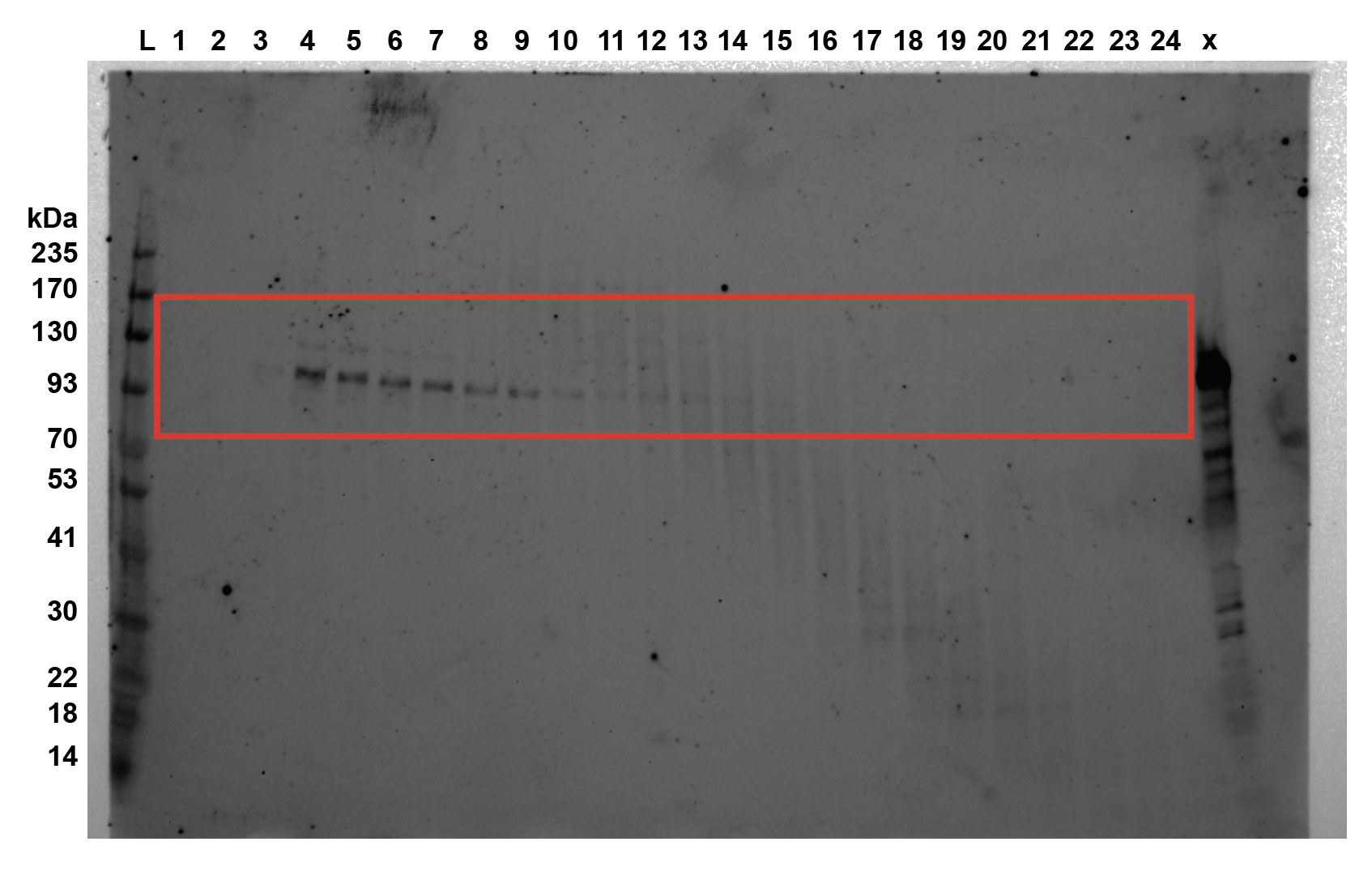

Supplement: Figure 5—figure supplement 1—source data 5. — Relevant bands for Figure 5—figure supplement 1C are highlighted with a red rectangle. L=ladder, in = input, x=lanes not used in Figure 5—figure supplement 1C. [file elife-83810-fig5-figsupp1-data5.zip › Figure 5-figure supplement 1-source data 5/Figure 5-figure supplement 1-source data 5 TAF4 annotated.tiff]

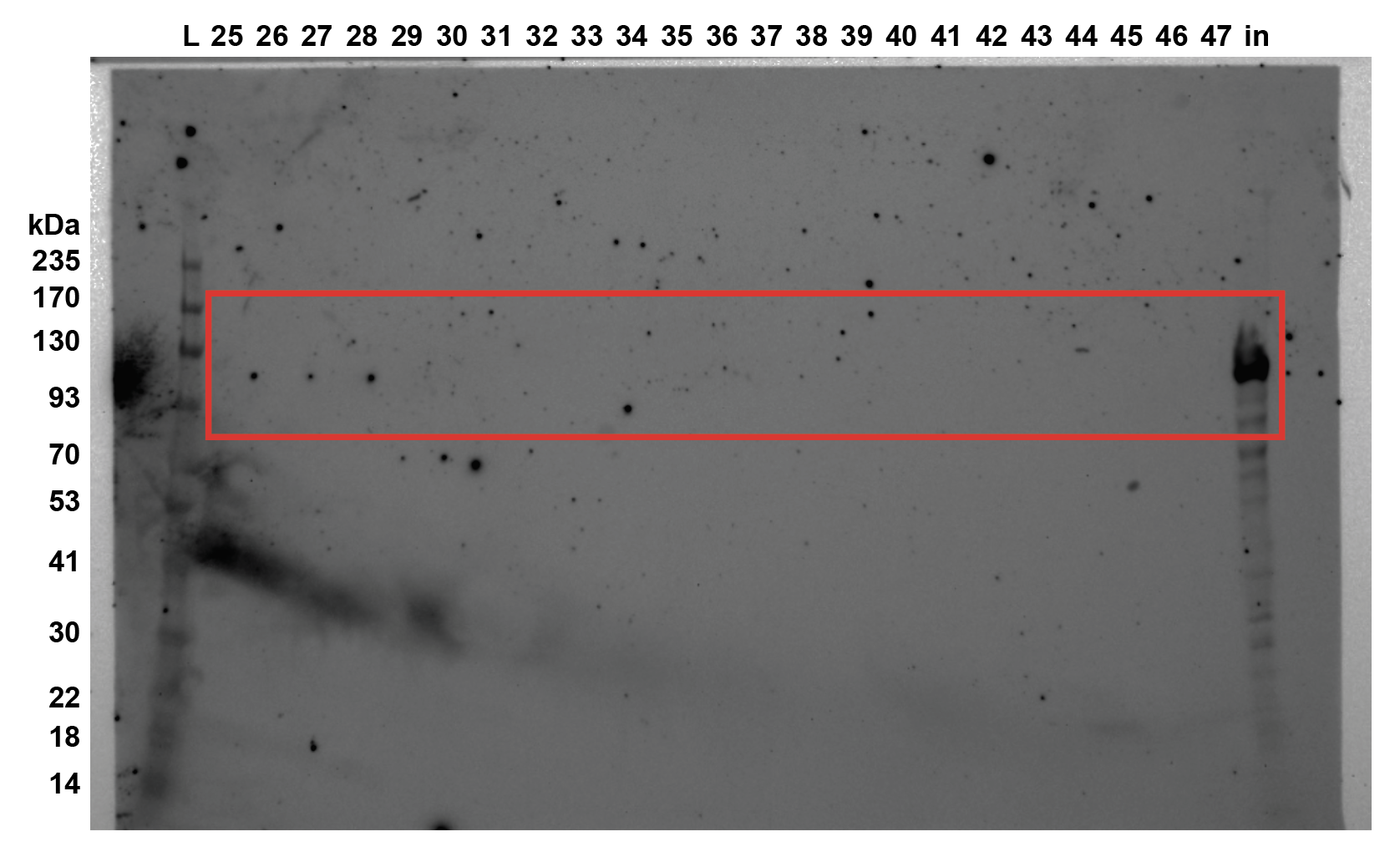

Supplement: Figure 5—figure supplement 1—source data 6. — Relevant bands for Figure 5—figure supplement 1C are highlighted with a red rectangle. L=ladder, in = input, x=lanes not used in Figure 5—figure supplement 1C. [file elife-83810-fig5-figsupp1-data6.zip › Figure 5-figure supplement 1-source data 6/Figure 5-figure supplement 1-source data 6 TAF4 annotated.tiff]

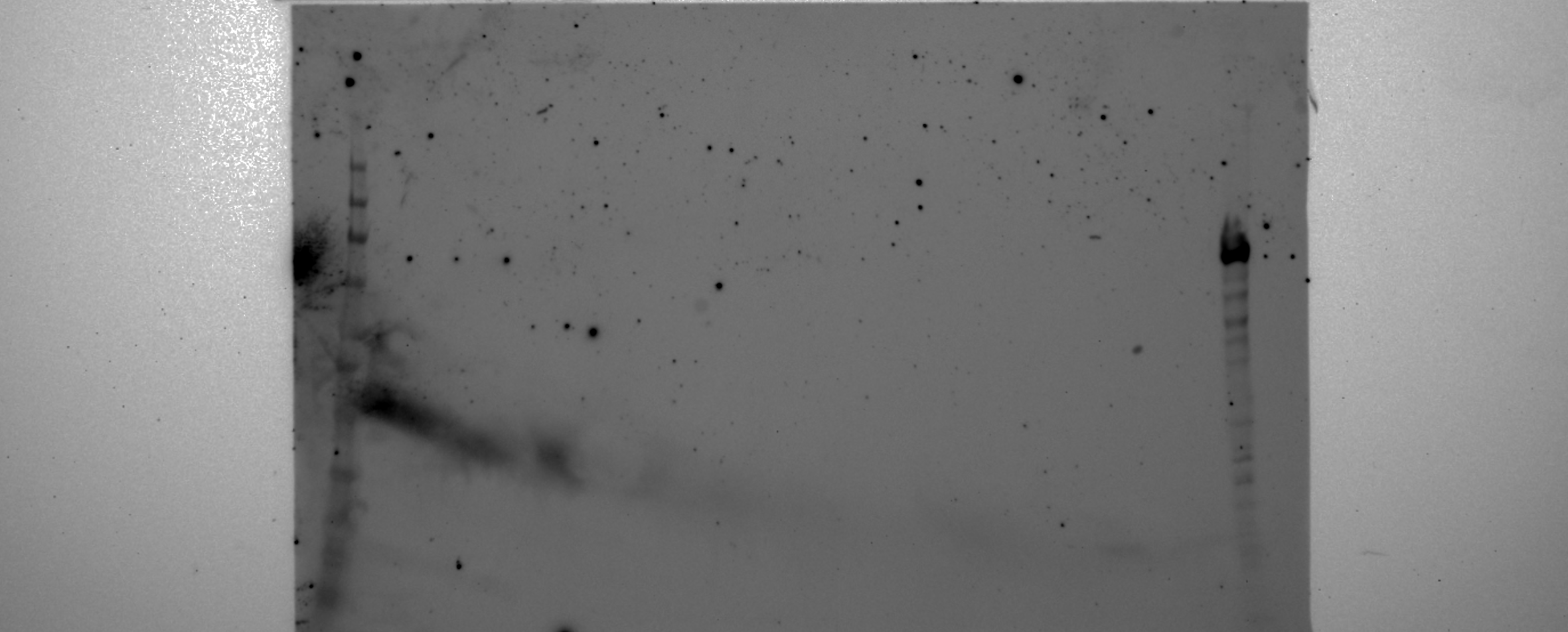

Supplement: Figure 5—figure supplement 1—source data 6. — Relevant bands for Figure 5—figure supplement 1C are highlighted with a red rectangle. L=ladder, in = input, x=lanes not used in Figure 5—figure supplement 1C. [file elife-83810-fig5-figsupp1-data6.zip › Figure 5-figure supplement 1-source data 6/Figure 5-figure supplement 1-source data 6 TAF4 raw.tif]

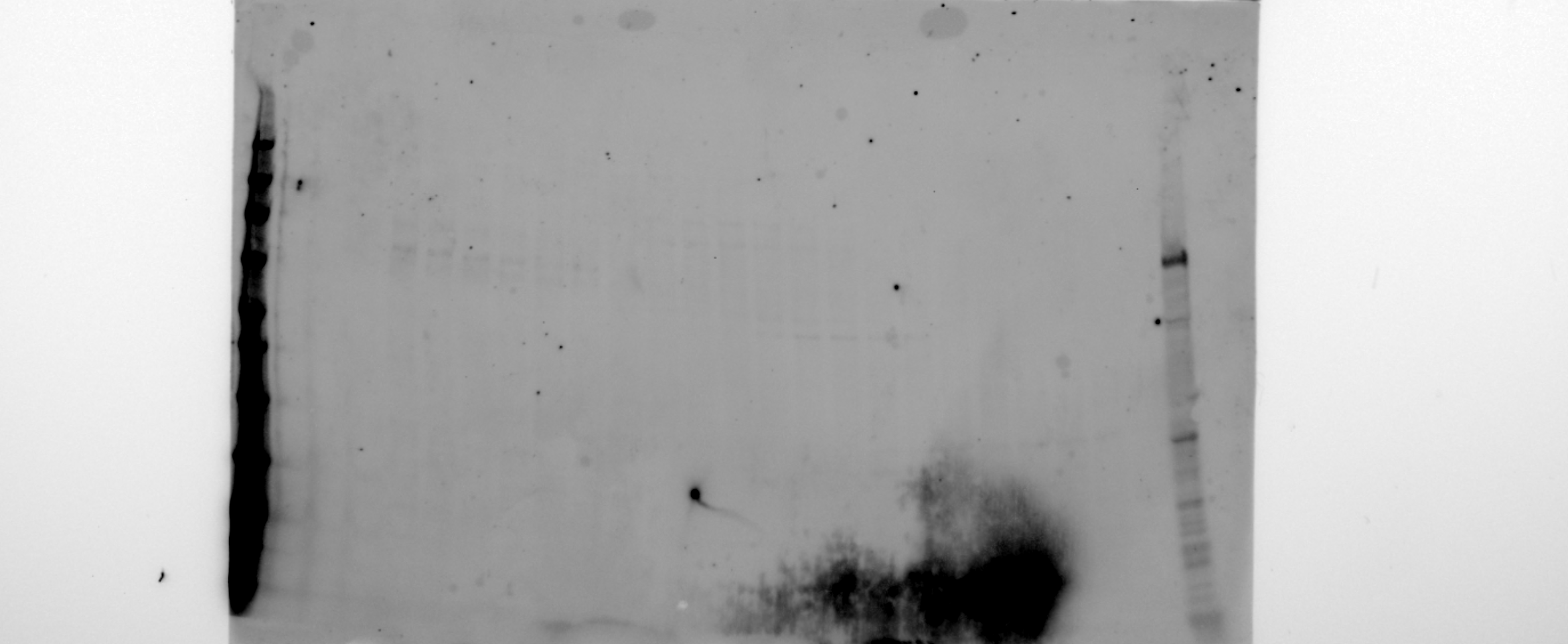

Supplement: Figure 5—figure supplement 1—source data 7. — Relevant bands for Figure 5—figure supplement 1C are highlighted with a red rectangle. L=ladder, in = input, x=lanes not used in Figure 5—figure supplement 1C. [file elife-83810-fig5-figsupp1-data7.zip › Figure 5-figure supplement 1-source data 7/Figure 5-figure supplement 1-source data 7 TBP raw.tif]

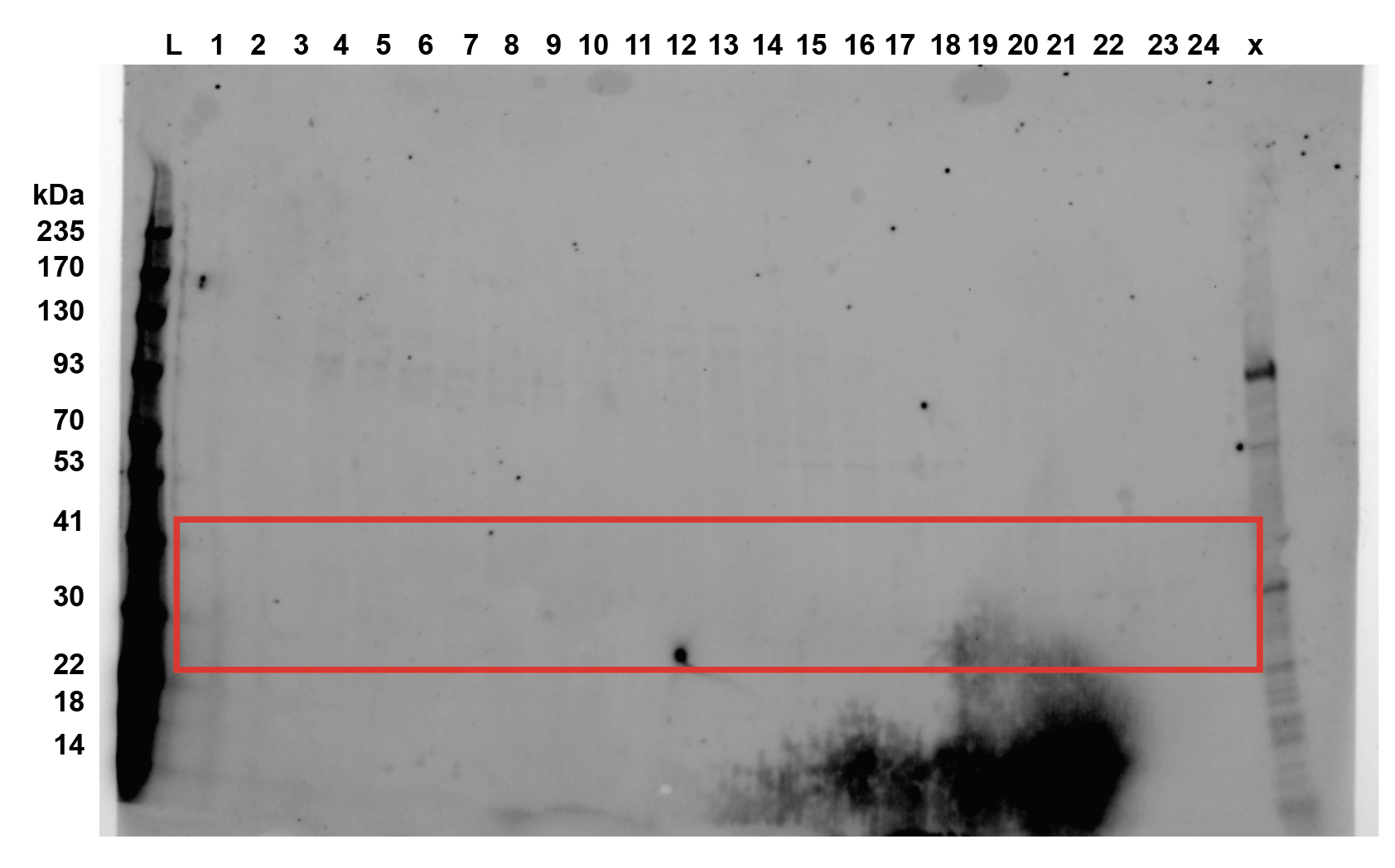

Supplement: Figure 5—figure supplement 1—source data 7. — Relevant bands for Figure 5—figure supplement 1C are highlighted with a red rectangle. L=ladder, in = input, x=lanes not used in Figure 5—figure supplement 1C. [file elife-83810-fig5-figsupp1-data7.zip › Figure 5-figure supplement 1-source data 7/Figure 5-figure supplement 1-source data 7 TBP annotated.tiff]

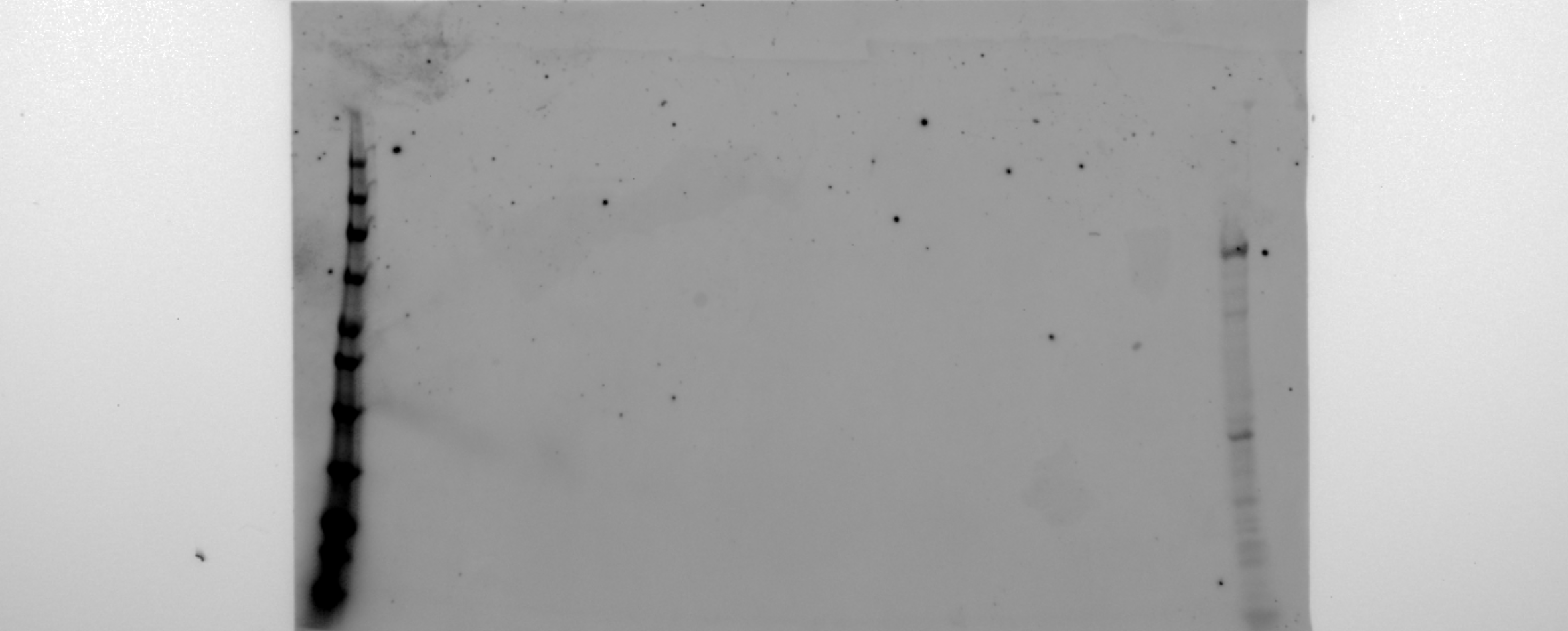

Supplement: Figure 5—figure supplement 1—source data 8. — Relevant bands for Figure 5—figure supplement 1C are highlighted with a red rectangle. L=ladder, in = input, x=lanes not used in Figure 5—figure supplement 1C. [file elife-83810-fig5-figsupp1-data8.zip › Figure 5-figure supplement 1-source data 8/Figure 5-figure supplement 1-source data 8 TBP raw.tif]

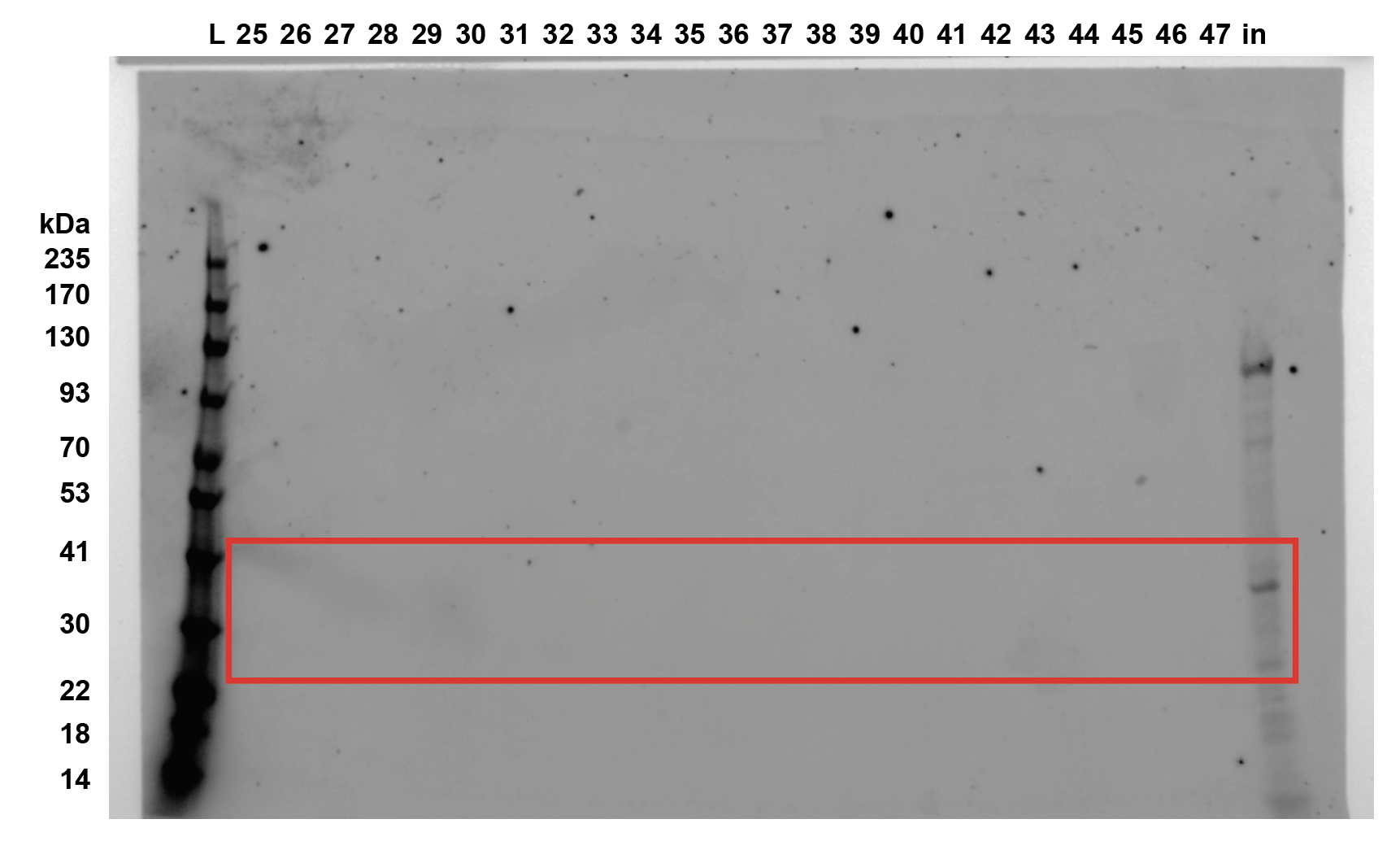

Supplement: Figure 5—figure supplement 1—source data 8. — Relevant bands for Figure 5—figure supplement 1C are highlighted with a red rectangle. L=ladder, in = input, x=lanes not used in Figure 5—figure supplement 1C. [file elife-83810-fig5-figsupp1-data8.zip › Figure 5-figure supplement 1-source data 8/Figure 5-figure supplement 1-source data 8 TBP annotated.tiff]

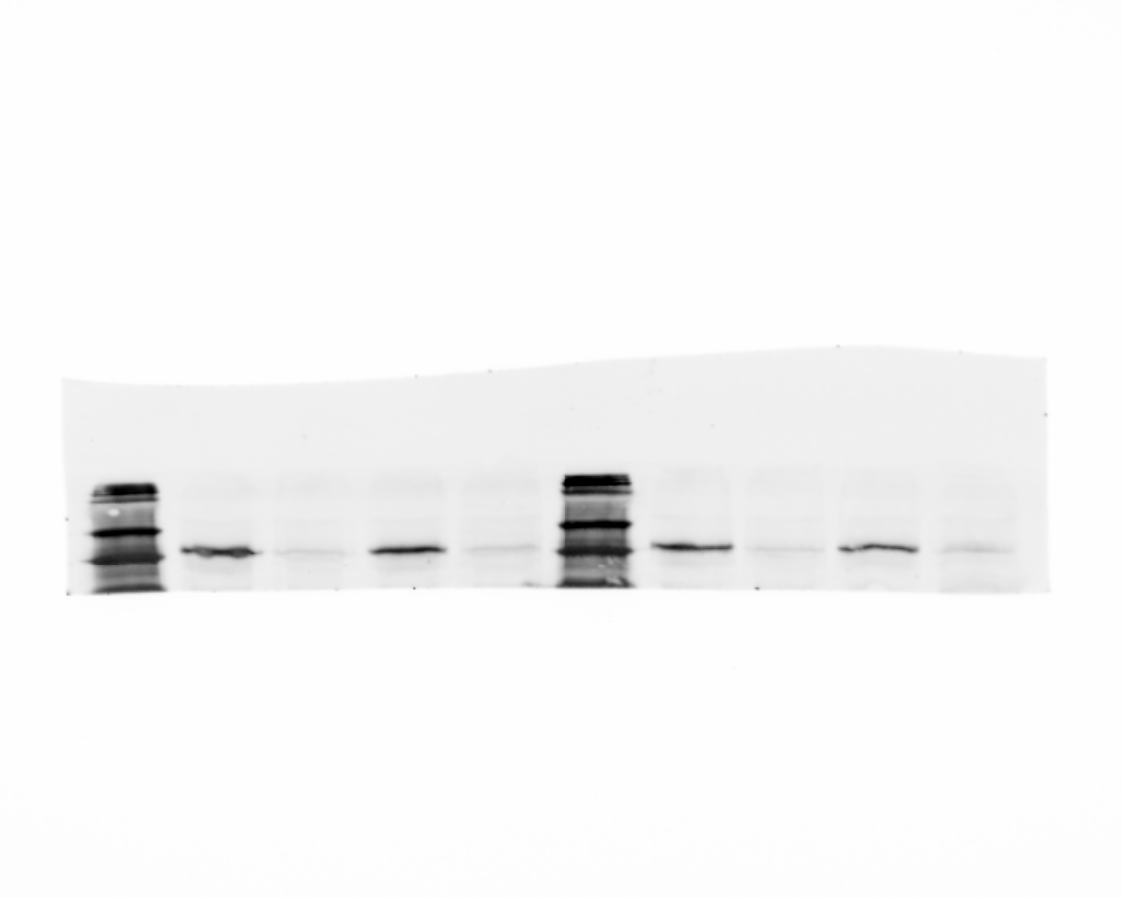

Supplement: Figure 6—figure supplement 2—source data 1. — Relevant bands for Figure 6—figure supplement 2A are highlighted with a red rectangle. L=ladder, x=lanes not used in Figure 6—figure supplement 2A. [file elife-83810-fig6-figsupp2-data1.zip › Figure 6-figure supplement 2-source data 1/Figure 6-figure supplement 2-source data 1 TBP raw.tif]

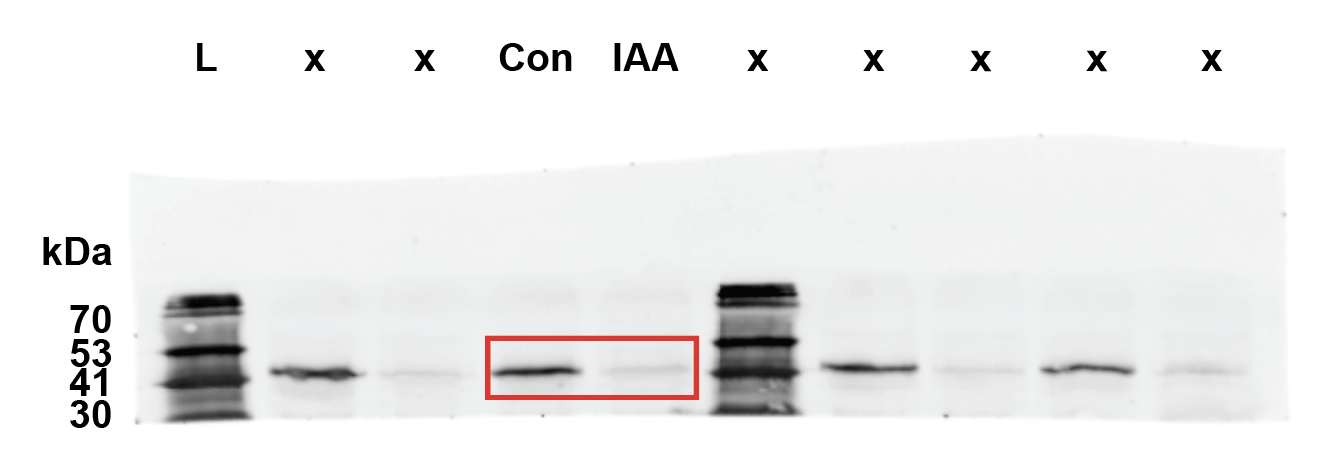

Supplement: Figure 6—figure supplement 2—source data 1. — Relevant bands for Figure 6—figure supplement 2A are highlighted with a red rectangle. L=ladder, x=lanes not used in Figure 6—figure supplement 2A. [file elife-83810-fig6-figsupp2-data1.zip › Figure 6-figure supplement 2-source data 1/Figure 6-figure supplement 2-source data 1 TBP annotated.tiff]

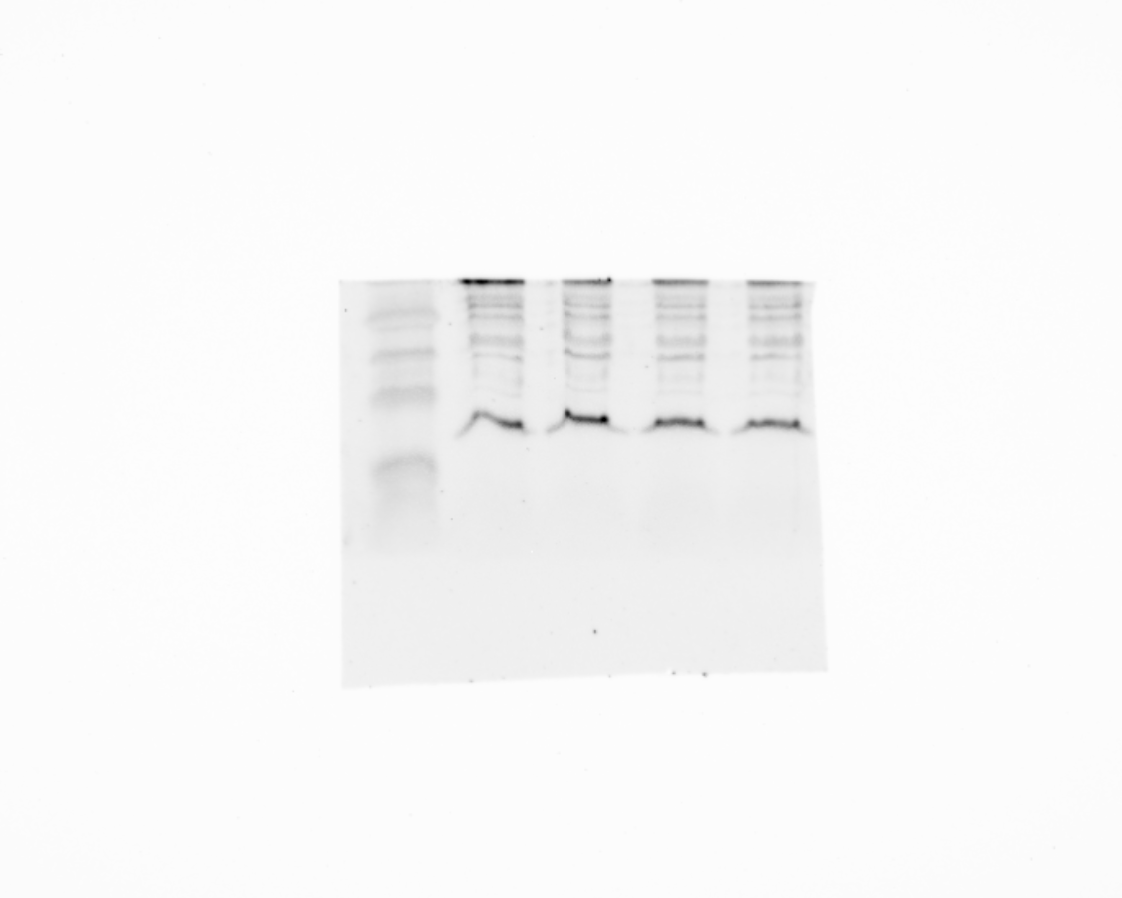

Supplement: Figure 6—figure supplement 2—source data 2. — Relevant bands for Figure 6—figure supplement 2A are highlighted with a red rectangle. L=ladder, x=lanes not used in Figure 6—figure supplement 2A. [file elife-83810-fig6-figsupp2-data2.zip › Figure 6-figure supplement 2-source data 2/Figure 6-figure supplement 2-source data 2 TFIIA raw.tif]

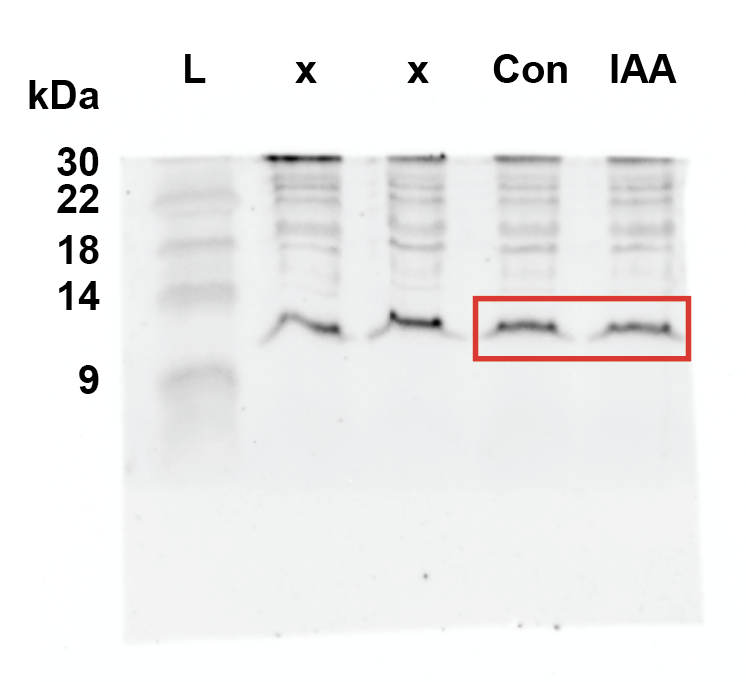

Supplement: Figure 6—figure supplement 2—source data 2. — Relevant bands for Figure 6—figure supplement 2A are highlighted with a red rectangle. L=ladder, x=lanes not used in Figure 6—figure supplement 2A. [file elife-83810-fig6-figsupp2-data2.zip › Figure 6-figure supplement 2-source data 2/Figure 6-figure supplement 2-source data 2 TFIIA annotated.tiff]

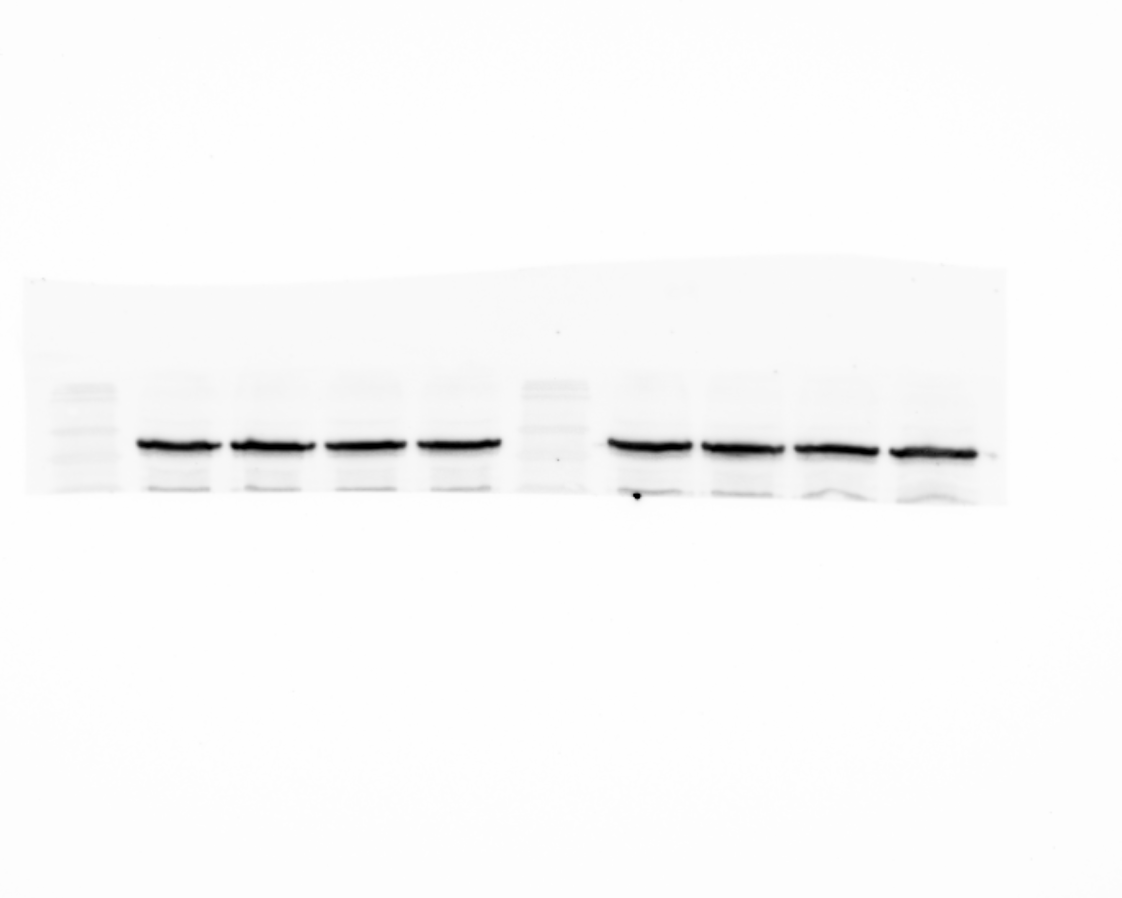

Supplement: Figure 6—figure supplement 2—source data 3. — Relevant bands for Figure 6—figure supplement 2A are highlighted with a red rectangle. L=ladder, x=lanes not used in Figure 6—figure supplement 2A. [file elife-83810-fig6-figsupp2-data3.zip › Figure 6-figure supplement 2-source data 3/Figure 6-figure supplement 2-source data 3 Tubulin raw.tif]

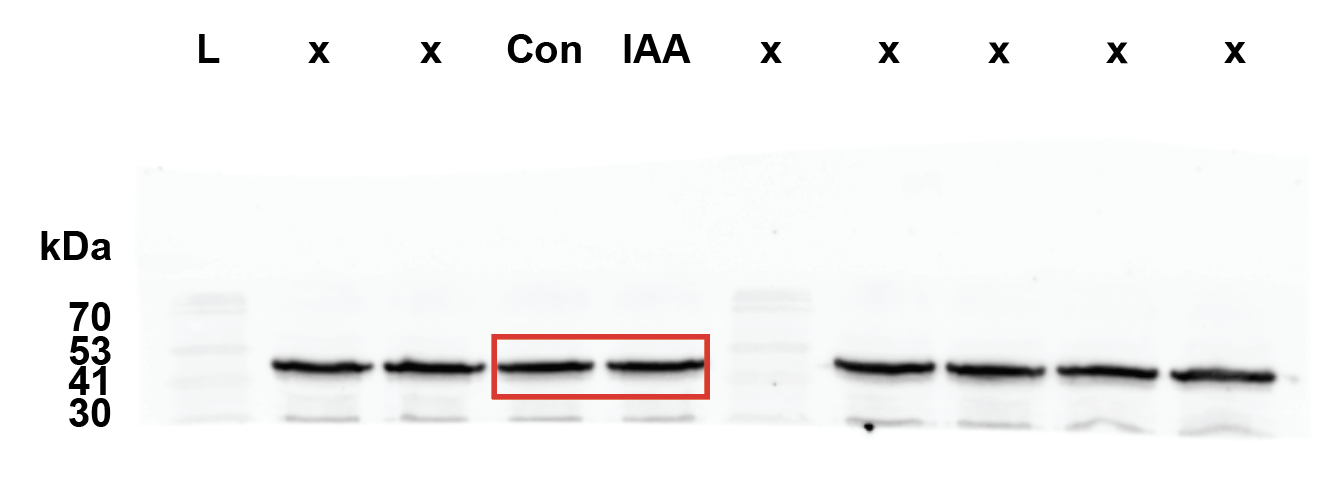

Supplement: Figure 6—figure supplement 2—source data 3. — Relevant bands for Figure 6—figure supplement 2A are highlighted with a red rectangle. L=ladder, x=lanes not used in Figure 6—figure supplement 2A. [file elife-83810-fig6-figsupp2-data3.zip › Figure 6-figure supplement 2-source data 3/Figure 6-figure supplement 2-source data 3 Tubulin annotated.tiff]
