# Supplementary material for: RNA Polymerase II transcription independent of TBP in murine embryonic stem cells
Source: eLife. 2023 Mar 30;12:e83810. doi: 10.7554/eLife.83810 (PMC10174690; doi:10.7554/eLife.83810)
Supplement: Supplementary file 1. [file elife-83810-supp1.docx]

**Supplementary File 1. Top upregulated/downregulated genes extracted from DGE analysis of Pol II CUT&Tag for all genes in control and IAA-treated C64 mESCs based on Figure 1F.**

| **Geneid** | **logFC** | **logCPM** | **LR** | **PValue** | **FDR** |
| --- | --- | --- | --- | --- | --- |
| *1110038B12Rik* | -2.579 | 5.503 | 53.209 | 3.00E-13 | 9.69E-10 |
| *Dipk1b* | -2.031 | 5.918 | 79.327 | 5.26E-19 | 2.84E-15 |
| *Snora43* | -1.970 | 2.703 | 18.017 | 2.19E-05 | 1.54E-02 |
| *Hspa8* | -1.732 | 8.459 | 82.276 | 1.18E-19 | 9.56E-16 |
| *Pabpc4* | -1.676 | 7.775 | 87.493 | 8.46E-21 | 1.37E-16 |
| *Rps5* | -1.580 | 5.963 | 41.417 | 1.23E-10 | 2.84E-07 |
| *Rab26* | -1.449 | 6.263 | 56.836 | 4.74E-14 | 1.91E-10 |
| *Snx5* | -1.322 | 9.409 | 49.527 | 1.96E-12 | 5.27E-09 |
| *Lrrc75a* | -1.318 | 9.644 | 16.555 | 4.73E-05 | 2.63E-02 |
| *Rps9* | -1.255 | 6.491 | 35.465 | 2.60E-09 | 4.66E-06 |
| *2700038G22Rik* | -1.220 | 5.533 | 24.927 | 5.95E-07 | 5.80E-04 |
| *Ahcy* | -1.198 | 5.421 | 28.209 | 1.09E-07 | 1.26E-04 |
| *Klhl13* | -1.186 | 5.755 | 33.568 | 6.88E-09 | 1.11E-05 |
| *Rnf225* | -1.126 | 5.407 | 28.745 | 8.25E-08 | 1.03E-04 |
| *Rabggtb* | -1.110 | 6.522 | 17.580 | 2.75E-05 | 1.85E-02 |
| *Slc35b2* | -1.106 | 8.229 | 37.211 | 1.06E-09 | 2.14E-06 |
| *Rfc4* | -1.083 | 6.947 | 32.387 | 1.26E-08 | 1.86E-05 |
| *Hsp90ab1* | -1.077 | 8.194 | 26.102 | 3.24E-07 | 3.49E-04 |
| *Pabpc1* | -1.026 | 7.423 | 30.767 | 2.91E-08 | 3.92E-05 |
| *Odc1* | -1.012 | 6.326 | 16.995 | 3.75E-05 | 2.24E-02 |
| *Rbm3* | -0.997 | 6.642 | 24.879 | 6.11E-07 | 5.80E-04 |
| *Eif4a2* | -0.985 | 7.864 | 17.311 | 3.17E-05 | 2.05E-02 |
| *Eef1b2* | -0.957 | 6.573 | 18.450 | 1.74E-05 | 1.28E-02 |
| *2410006H16Rik* | -0.930 | 5.915 | 19.894 | 8.19E-06 | 6.96E-03 |
| *P4hb* | -0.858 | 7.442 | 18.979 | 1.32E-05 | 1.07E-02 |
| *Nap1l1* | -0.843 | 8.632 | 17.035 | 3.67E-05 | 2.24E-02 |
| *Tpt1* | -0.827 | 6.780 | 20.030 | 7.62E-06 | 6.84E-03 |
| *Tlcd1* | -0.773 | 7.527 | 18.745 | 1.49E-05 | 1.15E-02 |
| *Sqstm1* | -0.726 | 7.848 | 16.923 | 3.89E-05 | 2.25E-02 |
