# Supplementary material for: RNA Polymerase II transcription independent of TBP in murine embryonic stem cells
Source: eLife. 2023 Mar 30;12:e83810. doi: 10.7554/eLife.83810 (PMC10174690; doi:10.7554/eLife.83810)
Supplement: Supplementary file 2. [file elife-83810-supp2.docx]

**Supplementary File 2. Raw values of a few top upregulated HS genes extracted from DGE analysis of Pol II CUT&Tag on all genes in control vs. HS-treated C64 mESCs.**

| **Geneid** | **logFC** | **logCPM** | **LR** | **Pvalue** | **FDR** |
| --- | --- | --- | --- | --- | --- |
| *Hsph1* | 4.062 | 9.616 | 179.026 | 7.91E-41 | 1.29E-36 |
| *Hspa1a* | 3.278 | 3.673 | 33.786 | 6.15E-09 | 2.51E-06 |
| *Hspa1b* | 2.814 | 5.340 | 69.229 | 8.77E-17 | 3.58E-13 |
| *Hspa4l* | 1.974 | 5.810 | 47.590 | 5.25E-12 | 7.15E-09 |
| *Dnajb1* | 1.807 | 5.739 | 29.966 | 4.40E-08 | 1.44E-05 |
| *Dnaja1* | 1.409 | 7.753 | 30.129 | 4.04E-08 | 1.40E-05 |
| *Dnajb13* | 1.233 | 5.385 | 19.648 | 9.31E-06 | 8.35E-04 |
| *Hsp90aa1* | 1.199 | 8.503 | 18.535 | 1.67E-05 | 1.32E-03 |
| *Hspe1* | 1.145 | 5.267 | 9.793 | 1.75E-03 | 4.14E-02 |
| *Dnajc21* | 1.073 | 6.428 | 17.202 | 3.36E-05 | 2.22E-03 |
