# Supplementary material for: RNA Polymerase II transcription independent of TBP in murine embryonic stem cells
Source: eLife. 2023 Mar 30;12:e83810. doi: 10.7554/eLife.83810 (PMC10174690; doi:10.7554/eLife.83810)
Supplement: Supplementary file 3. [file elife-83810-supp3.docx]

**Supplementary File 3. Gene ontology analysis of the top upregulated genes extracted from DGE analysis of Pol II CUT&Tag in control vs. HS-treated C64 mESCs from Figure 1-figure supplemental 3A.**

| **GO molecular function complete** | **REF LIST** | **Input LIST** | **Expected** | **fold Enrichment** | **P-value** | **FDR** |
| --- | --- | --- | --- | --- | --- | --- |
| C3HC4-type RING finger domain binding | 6 | 3 | 0.07 | 41.35 | 1.34E-04 | 2.72E-02 |
| DNA replication origin binding | 15 | 5 | 0.18 | 27.57 | 3.14E-06 | 2.54E-03 |
| single-stranded DNA helicase activity | 23 | 5 | 0.28 | 17.98 | 1.84E-05 | 6.38E-03 |
| protein folding chaperone | 41 | 6 | 0.5 | 12.1 | 1.96E-05 | 6.33E-03 |
| unfolded protein binding | 90 | 9 | 1.09 | 8.27 | 2.94E-06 | 3.56E-03 |
| single-stranded DNA binding | 122 | 10 | 1.48 | 6.78 | 4.35E-06 | 3.02E-03 |
| chaperone binding | 112 | 9 | 1.35 | 6.65 | 1.53E-05 | 6.73E-03 |
| heat shock protein binding | 142 | 9 | 1.72 | 5.24 | 8.74E-05 | 2.12E-02 |
| integrin binding | 154 | 9 | 1.86 | 4.83 | 1.57E-04 | 2.92E-02 |
| cell adhesion molecule binding | 306 | 13 | 3.7 | 3.51 | 1.31E-04 | 2.90E+00 |
