# Supplementary material for: RNA Polymerase II transcription independent of TBP in murine embryonic stem cells
Source: eLife. 2023 Mar 30;12:e83810. doi: 10.7554/eLife.83810 (PMC10174690; doi:10.7554/eLife.83810)
Supplement: Supplementary file 4. [file elife-83810-supp4.docx]

**Supplementary File 4. Raw values of a few top upregulated RA genes extracted from edgeR DGE analysis of Pol II CUT&Tag on all genes in control vs. RA-treated C64 mESCs**

| **Geneid** | **logFC** | **logCPM** | **LR** | **Pvalue** | **FDR** |
| --- | --- | --- | --- | --- | --- |
| *Stra8* | 3.766 | 6.940 | 109.154 | 1.50E-25 | 1.23E-21 |
| *Nrip1* | 2.657 | 7.102 | 83.134 | 7.67E-20 | 4.18E-16 |
| *Pbx1* | 2.167 | 7.973 | 50.153 | 1.42E-12 | 4.65E-09 |
| *Rbp1* | 1.899 | 4.396 | 27.580 | 1.51E-07 | 1.37E-04 |
| *Islr* | 1.548 | 4.947 | 26.093 | 3.25E-07 | 2.31E-04 |
| *Cdx1* | 1.293 | 4.588 | 14.064 | 1.77E-04 | 2.71E-02 |
| *Zfyve28* | 1.287 | 7.246 | 22.712 | 1.88E-06 | 8.55E-04 |
| *Steap3* | 1.281 | 6.480 | 21.198 | 4.14E-06 | 1.54E-03 |
| *Igsf21* | 1.278 | 8.550 | 24.145 | 8.93E-07 | 4.30E-04 |
| *Tspan9* | 1.248 | 8.193 | 16.591 | 4.64E-05 | 9.35E-03 |
