# Supplementary material for: RNA Polymerase II transcription independent of TBP in murine embryonic stem cells
Source: eLife. 2023 Mar 30;12:e83810. doi: 10.7554/eLife.83810 (PMC10174690; doi:10.7554/eLife.83810)
Supplement: Supplementary file 5. — Oligonucleotides were modified with the addition of a 5’ biotin group and purified by HPLC. [file elife-83810-supp5.docx]

**Supplementary File 5. Biotinylated snRNA, snoRNA, lncRNA, and rRNA depletion oligonucleotides for NET-seq library construction.**

Oligonucleotides were modified with the addition of a 5’ biotin group and purified by HPLC.

| **Gene** | **Type** | **Sequence** |
| --- | --- | --- |
| *Rn45s* | rRNA | GCCTGGCGCGGCTTAGGCCCTGGCCCGAAGAGAACTCCGGAG |
| *Gm22265* | snRNA | GCAAGAAGCAAGACCTCAAAAAATTGG |
| *Gm25099* | snRNA | GCACGAGCAGGACCTCAAAAAATTGGG |
| *Snord118* | snoRNA | ATAGGAGCAACCAGGATGTTGTCAGGTCCTGATTGCATGG |
| *Gm23287* | snRNA | GTAAGCACAAGACCTCAAAAAATTGGGTTAAGACTC |
| *Gm23143* | snRNA | AGTCCTGGGTAGGCAGGACCTCAAAAAATTGAGTTAAGACTC |
| *Snord75* | snoRNA | AAGCCTCAGAAAAGTGTTTTTCAGAAATCCC |
| *Snord30* | snoRNA | AATCAGATTTCCAAGTCTCAACAGCAATCATCAGCCG |
| *Snord2* | snoRNA | GATCAGCAAGAGTATTCTCTTCATTTCAGGTC |
| *LOC115487645* | snoRNA | TCAGATAGGAGCGAAAGACATGATTGTTCATC |
| *Snord60* | snoRNA | GCCTCAGTCTTATAATGTAATCAGACTACGC |
| *Snord3a* | snoRNA | ACTCAGACTGTGTCCTCTCCCTCTCAACCCTCAAGAG |
| *Snord74* | snoRNA | ATCAGAATCGTTGGTATTCATCAACAAGCTCAG |
| *Snord27* | snoRNA | TTCAGTAGTAAGATGACATCATTGCAACTCAGCCATATGC |
| *Snord25* | snoRNA | TCCTCAGAGTTACTTATCCTCACAGATATATCAGTACAGG |
| *Rnu5g* | snRNA | GTCAAGACAAGGCCTCAAAAAATTGG |
| *Gm25313* | snRNA | GCCGAAACAAGGCCTCAAAAAATTGG |
| *LOC115487699* | snRNA | GCTAAAGCAAGGCCTCAAAAAATTAGT |
| *Snord43* | snoRNA | AAATCAGAACTTGACATTCAGCACAGAGTTTCTGT |
| *Snord13* | snoRNA | TGTCAGACGGGTAATGTGCCCACGTCGTAACAAGG |
| *Snord52* | snoRNA | TGAGTCAGATTTATAGCTGACATCAAGGACAG |
| *Snord83b* | snoRNA | TCTCAGAAGGAAGGCAACAGGGAATAATTCCTCAG |
| *Rps20* | rRNA | TCAGAATAGCGTATAAGAAATATCAC |
| *Rnu7* | snRNA | AGGGGTTTTCCGACCGAAGTCAGAAAACC |
| *1110038B12Rik* | lncRNA | TGGGTCAGTTACAAGGAGTGGCGTCACGTGG |
| *Snord4a* | snoRNA | CAATAGATCACAATCATCTTTATCAGAC |
| *Snord45b* | snoRNA | TCTCAGTGTAATTTGTAACTTGCATCAGGTTAGCATTC |
| *Snord10* | snoRNA | TGCTCTCAGAATACAAGGACTGATCC |
| *Snord45* | snoRNA | GGTCTCAGCGTAATTCTAGAGCTAAAGTG |
| *Snord42a* | snoRNA | AGTGGTTCCTTTGTTGGTGTCATTCTTTTCCAAACATTGAG |
