# Supplementary material for: RNA Polymerase II transcription independent of TBP in murine embryonic stem cells
Source: eLife. 2023 Mar 30;12:e83810. doi: 10.7554/eLife.83810 (PMC10174690; doi:10.7554/eLife.83810)
Supplement: Supplementary file 6. [file elife-83810-supp6.docx]

**Supplementary File 6. Primers used to knock-out *Tbpl1*, confirm *Tbpl1* knock-out and to test *Gapdh* mRNA levels via qRT-PCR.**

| **Gene Name** | **Sequence (5’ -> 3’)** | **Purpose** |
| --- | --- | --- |
| *Tbpl1* | CACCGATCACTGTCTGCATCCATTG | gRNA *Tbpl1* knock out forward #1 |
| *Tbpl1* | AAACCAATGGATGCAGACAGTGATC | gRNA *Tbpl1* knock out reverse #1 |
| *Tbpl1* | CACCGCAGGTCTCAAACGGTGCTC | gRNA *Tbpl1* knock out forward #2 |
| *Tbpl1* | AAACGAGCACCGTTTGAGACCTGC | gRNA *Tbpl1* knock out reverse #2 |
| *Tbpl1* | AGATTGCTTTGGAGGGAGCA | qRT-PCR screen *Tbpl1* knock out |
| *Tbpl1* | CCTGAGGACCAAATTGTAGCTG | qRT-PCR screen *Tbpl1* knock out |
| *Gapdh* Intron | TTCTGATCTCAGCTCCCCTG | quantify *Gapdh* levels or used to normalize |
| *Gapdh* Intron | GGCAACAATCTCCACTTTGC | quantify *Gapdh* levels or used to normalize |
| *Gapdh* Exon | CGGGTTCCTATAAATACGGACTG | quantify *Gapdh* levels |
| *Gapdh* Exon | CCAATACGGCCAAATCCGTTC | quantify *Gapdh* levels |
| *Rps6* | CGATATCCTCGGTGACGAGT | used to normalize S2 cells |
| *Rps6* | CCCTTCTTCAAGACGACCAG | used to normalize S2 cells |
