# Supplementary material for: RNA Polymerase II transcription independent of TBP in murine embryonic stem cells
Source: eLife. 2023 Mar 30;12:e83810. doi: 10.7554/eLife.83810 (PMC10174690; doi:10.7554/eLife.83810)
Supplement: Supplementary file 7. [file elife-83810-supp7.docx]

**Supplementary File 7. Primers used to test TBP depletion via ChIP-qPCR**

| **Gene Name** | **Sequence (5’ -> 3’)** | **Purpose** |
| --- | --- | --- |
| *Gapdh* | CCTATCAGTTCGGAGCCCAC | qPCR, forward |
| *Gapdh* | AGCTACGTGCACCCGTAAAG | qPCR, reverse |
| *Hspa1a* | GTAGCTGTCAGCGTCTGGTG | qPCR, forward |
| *Hspa1a* | CGATTACTCAAGGGAGGCGG | qPCR, reverse |
